# Cascaded YOLO-based approach for Bangladeshi license plate identification and number recognition

**DOI:** 10.1371/journal.pone.0355858

**Published:** 2026-08-10

**Authors:** Proshenjit Sarker, S. M. Shamsul Alam

**Affiliations:** Electronics and Communication Engineering Discipline, Khulna University, Khulna, Bangladesh; Kocaeli University: Kocaeli Universitesi, TÜRKIYE

## Abstract

Automatic vehicle license plate detection and recognition has become an important component in intelligent transportation systems, traffic monitoring, and law enforcement applications. However, existing approaches for Bangladeshi license plates often rely on optical character recognition (OCR) or recognition models with a limited number of character classes, which restricts their ability to handle the complex structure and large variation of Bangla license plate components. To address these challenges, this study proposes a vehicle license plate detection and recognition (VLPDR) framework based on a cascading architecture of three YOLOv12 models. The first model detects vehicles, the second model localizes the license plate region, and the third model performs character-level recognition without relying on conventional OCR engines. The proposed recognition model incorporates 104 classes, covering a wide range of Bangladeshi license plate components, including city codes, vehicle types, and numeric digits. Two datasets consisting of 9,357 images have been used for training, validation, and testing, while an additional 150 images have been used for external validation. The license plate detection (LPD) model has achieved testing precision, recall, and mAP@50 of 0.986, 0.965, and 0.963, respectively. The license plate number recognition (LPNR) model has obtained testing precision, recall, and mAP@50 values of 0.95, 0.898, and 0.949. During external validation, the cascaded framework achieved 100% license plate detection accuracy, 95.33% full-number recognition accuracy, 98.82% character-wise mean accuracy, and 99.57% Levenshtein-based similarity accuracy. The system has also been evaluated on multiple hardware platforms, demonstrating inference times of 2311.53 ms on a CPU-based laptop and 284.31 ms on a GPU-enabled computer. These results indicate that the proposed cascaded YOLO-based framework provides an effective and practical solution for Bangladeshi license plate detection and recognition with improved class coverage and strong generalization capability.

## 1. Introduction

**Fig 1:**
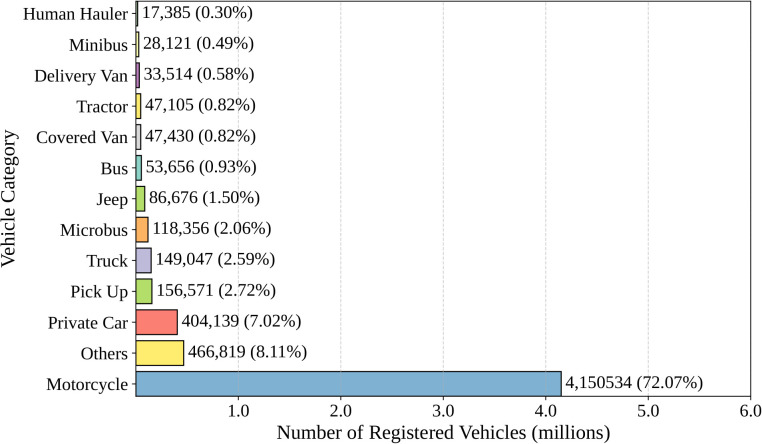
Registered vehicles in Bangladesh as of June 30, 2023 [4].

Automatic Vehicle License Number (AVLN) detection and recognition is a computer vision–based process that automatically localizes a vehicle’s license plate in an image or video frame and accurately extracts and interprets its alphanumeric characters to determine the vehicle’s registration number [1,2]. According to the World Health Organization, Bangladesh’s population exceeded 171 million in 2023 [3]. Due to rapid population growth, the demand for vehicles has increased significantly; as of June 30, 2023, the Bangladesh Road Transport Authority (BRTA) reported a cumulative total of 5,759,353 registered motor vehicles in Bangladesh, with motorcycles accounting for the largest share at over 4.1 million units (72.07% of total registered vehicle) [4], as visualized in Fig 1.

**Fig 2:**
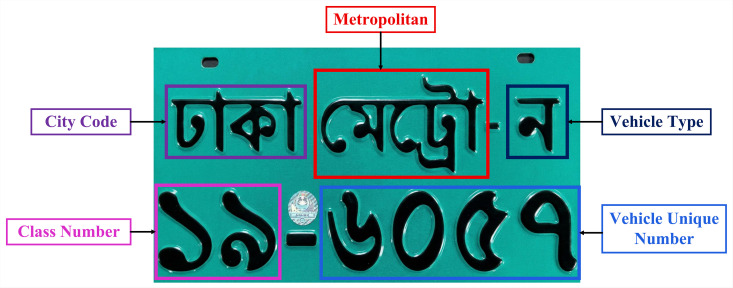
Standard Bangladeshi vehicle license plate description [4,5].

This substantial growth in vehicle density has intensified traffic congestion, road safety risks, and enforcement complexities. According to the Bangladesh Road Transport Authority (BRTA) Annual Report 2022–2023, vehicle-related violations are increasing due to illegal and unfit vehicles, unlicensed or expired driving licenses, overloading, over-speeding, and fare overcharging in public transport, leading to 13,128 cases and fines totaling 45,281,300 Taka across 2,160 mobile court operations [4]. These escalating challenges underscore the urgent need for AVLN detection and recognition systems to facilitate efficient traffic monitoring, automated enforcement, toll collection, parking automation, and enhanced public security in Bangladesh. A standard Bangladeshi Vehicle License Plate (BVLP) comprises four mandatory components: a City Code, a Vehicle Type Code (typically a Bengali letter), a two-digit series or class number, and a four-digit unique vehicle registration number [4,5]. Furthermore, vehicles registered within metropolitan jurisdictions feature an additional “Metro” designation immediately following the city code to indicate their specific registration area, as shown in Fig 2.

In recent years, machine learning (ML), particularly deep learning, has emerged as the most effective and widely adopted approach for AVLN detection and recognition. Over the past few decades, numerous ML-based computer vision algorithms have been developed, many of which are highly specialized and well-suited for this task. Convolutional Neural Networks (CNNs [6]) have significantly improved feature extraction and classification accuracy. In contrast, region-based detectors such as Faster Region-Based Convolutional Neural Network (Faster R-CNN [7]) and single-stage detectors like You Only Look Once (YOLO [8]) and Single Shot MultiBox Detector (SSD [9]) have enabled real-time and high-precision license plate localization. For character recognition, models such as Convolutional Recurrent Neural Network (CRNN [10]) and Long Short-Term Memory (LSTM [11]) networks have demonstrated strong sequential learning capability, making them highly effective for extracting alphanumeric characters from detected plates. These advancements have substantially enhanced the accuracy, robustness, and real-time performance of modern systems.

Over the last few years, these ML algorithms have been used globally, including in Bangladesh. A comprehensive review paper, [12], analyzed over 160 research papers and 40 datasets from 18 countries (including China, Brazil, Tunisia, France, Germany, and the USA) published since 2015, examining more than 30 distinct deep learning-based algorithms for license plate detection (such as YOLO variants and VertexNet) and over 20 recognition methods (including connectionist temporal classification-based and attention-based approaches). Another paper, [13], by Imtiaz et al. proposed a specialized Automatic Number Plate Recognition (ANPR) system for Category L vehicles (motorcycles, mopeds) designed for Remote Emission Sensing (RES). The Category L ANPR (L-ANPR) integrates photoelectric sensors for vehicle size detection and a YOLOv4 detector trained on European plates, achieving over 90% detection accuracy, followed by Tesseract Optical Character Recognition (OCR) with about 70% recognition accuracy. Validated in Austria and tested in Belgium, it successfully identified plates from France, Italy, and Switzerland. Limitations due to low resolution and lighting were addressed with IR-illuminated cameras and AI-based recognition enhancements.

From the perspective of Bangladesh, we have identified multiple existing works that align with our work as summarized in Table 1, considering recent literature on character detection and recognition, detailing the specific activities, algorithmic approaches, number of character classes evaluated, and the resulting performance metrics. [14] achieved a detection accuracy of 97.7% using a Hybrid YCbCr color model and reached a recognition accuracy of 99.3% across 14 classes by combining a Convolutional Neural Network (CNN) for feature extraction with a Support Vector Machine (SVM) for classification. Similarly, [15] utilized Morphological Operations (MO) and Histogram analysis for Region of Interest (ROI) extraction, achieving 91.10% detection accuracy, followed by a Histogram of Oriented Gradients (HOG) combined with an SVM to achieve a flawless 100% recognition accuracy on 18 classes.

**Table 1:** Related previous work summary.

Ref.	Activity	Algorithms	Class	Performance	Disadvantage
[14]	Detection	Hybrid YCbCr		Detection Accuracy: 97.7%	Limited dataset size (1400) and class (14)
	Recognition	CNN + SVM	14	Recognition Accuracy: 99.3%	
[16]	Detection	YOLOv5		Detection Accuracy: 98%	OCR has failed to recognize conjunct consonants.
	Recognition	EasyOCR		Recognition Accuracy: 78%	
[17]	Detection	YOLOv4		Detection Accuracy: 90.50%	Dataset were created from a single video.
	Recognition	Tesseract version 4 OCR		Image-wise Accuracy: 22% – 73%.	
[15]	Detection	MO + Histogram		Detection (ROI Extraction): 91.10%	Less number of classes (18) were recognized.
	Recognition	HOG + SVM	18	Recognition Accuracy: 100%	
[18]	Detection	YOLOv4		Detection Accuracy: 99.37%	Limited number of classes (27) for recognition.
	Recognition	YOLOv4	27	Recognition Accuracy: 96.31%	
[19]	Detection	YOLOv4		Detection Accuracy: 99.89% mAP	Limited number of classes (30) for recognition.
	Recognition	Custom CNN	30	Recognition f1-score: 99.33%	
[20]	Detection	YOLOv7		Detection Accuracy: 91%	Limited number of classes (24) for recognition.
	Recognition	VGG19	24	Recognition Accuracy: 93.69%	
[21]	Detection	Extended Inception-v3		Detection MSE: 0.015	Only 60 classes and utilization of OCR.
	Recognition	Custom CNN + OCR	60	Recognition Accuracy: 95%	
[5]	Detection	YOLOv7		Detection Accuracy: 96%	Only 60 classes and utilization of OCR.
	Recognition	Custom OCR + XGBoost	60	Recognition Accuracy: 97%	
[22]	Recognition	Custom CNN	17	Test Accuracy: 96%	Only 17 classes were recognized
[23]	Detection	YOLOv8		Mean mAP 0.934	No recognition task were performed.
[24]	Detection	YOLOv8		Detection Accuracy: 97.8%	Limited by the utilization of OCR.
	Recognition	OCR			
[25]	Detection	YOLOv10		Detection Accuracy: 96.87%	Low recognition accuracy.
	Recognition	DONUT model		Recognition Accuracy: 89.96%	
[26]	Detection	YOLOv9		Detection F-score: 99%	Limited by the utilization of OCR.
	Recognition	OCR		Recognition Accuracy: 99.97%	
[27]	Detection	YOLOv12		Detection Accuracy: 97.9%,	OCR-based performance was not properly reported.
	Recognition	EasyOCR		–	

The You Only Look Once (YOLO) family of models is widely used in detection tasks across multiple studies, especially for surveillance [28]. [16] paired YOLOv5 (98% detection accuracy) with EasyOCR for Optical Character Recognition (OCR), which yielded a 78% recognition accuracy. YOLOv4 was widely adopted: [17] combined it with Tesseract version 4 OCR, noting a 90.50% detection accuracy but highly variable image-wise recognition ranging from 22% to 73%; [18] used YOLOv4 for both detection (99.37%) and recognition (96.31% across 27 classes); and [19] recorded a 99.89% mean Average Precision (mAP) using YOLOv4 before applying a Custom CNN to reach a 99.33% f-score on 30 classes.

More recent iterations of YOLO have also been heavily explored. [20] utilized YOLOv7 for detection (91%) alongside Visual Geometry Group 19 (VGG19) for recognition (93.69% on 24 classes). Another YOLOv7 implementation by [5] achieved 96% detection and paired it with a Custom OCR and Extreme Gradient Boosting (XGBoost) classifier to recognize 60 classes with 97% accuracy. Ramit et al., [23], used YOLOv8, yielding a Mean mAP of 0.934 for detection, and by [24], which achieved 97.8% detection before an unspecified OCR step. [25] pushed into newer architectures, combining YOLOv10 (96.87% detection) with the Document Understanding Transformer (DONUT) model, realizing an 89.96% recognition accuracy. Finally, several authors focused on specialized or custom deep learning networks. [21] utilized an Extended Inception-v3 architecture for detection, resulting in a low Mean Squared Error (MSE) of 0.015, and a Custom CNN-based OCR for identifying 60 classes with 95% accuracy. Focusing strictly on the recognition phase, [22] deployed a Custom CNN to secure a 96% test accuracy across 17 distinct classes. [26] and [27] followed a comparable two-stage methodology, utilizing YOLOv9 and YOLOv12, respectively, for license plate detection, with OCR subsequently applied for character recognition.

Despite significant progress in Bangladeshi license plate detection and recognition, several limitations remain in existing studies. Many recent works have used YOLO-based models for both detection and recognition; however, the recognition stage often involves only a limited number of classes, typically ranging from 14 to 60. In practice, Bangladeshi license plates contain a much larger variety of character combinations, which can exceed 100 classes when considering digits, district names, and other textual components. This limited class coverage restricts the real-world applicability of such systems. Additionally, several studies rely on generic OCR engines for license plate recognition. While OCR-based approaches simplify the recognition pipeline, they often struggle with the complex structure of the Bangla script. In particular, Bangla includes conjunct consonants known as *Jukto Borno*, where two or more consonants combine to form a single ligature. These compound characters increase the visual complexity of the text and are frequently misrecognized by conventional OCR models, leading to reduced recognition accuracy in practical scenarios.

Given the existing research gaps, this work primarily focuses on avoiding OCR by replacing it with YOLO, a more advanced deep learning algorithm capable of both detection and recognition. Another major focus is to include more than 100 classes in the YOLO model to cover all components of Bangladeshi vehicle license plates, including city codes, vehicle types, and numeric digits. Therefore, the key contributions of this study can be summarized as follows:

- **Cascaded YOLOv12-Based VLPDR Framework:** This work proposes an advanced vehicle license plate detection and recognition (VLPDR) framework based on a cascaded architecture of three YOLOv12 models for end-to-end license plate localization and recognition.
- **Comprehensive 104-Class Recognition Scheme:** The proposed system introduces a 104-class recognition scheme that covers almost all categories of Bangladeshi license plate components, including city codes, vehicle types, and numeric digits, enabling more comprehensive recognition than existing approaches.
- **OCR-Free Recognition Strategy:** The proposed framework directly recognizes license plate information through object detection and classification without relying on OCR, thereby mitigating recognition errors caused by complex Bangla characters and conjunct consonants (*Jukto Borno*).
- **Robust Cascaded Recognition Pipeline:** The multi-stage cascading architecture reduces false detections and improves the robustness and accuracy of the overall recognition process.
- **Generalization Assessment Through External Validation:** Extensive experiments and external validation are conducted to evaluate the effectiveness and generalization capability of the proposed framework.
- **Levenshtein-Based Similarity Evaluation:** A Levenshtein distance-based similarity analysis is performed to quantify the correspondence between recognized and ground-truth license plate numbers.
- **Edge Deployment on NVIDIA Jetson:** The proposed framework is deployed and tested on an NVIDIA Jetson developer kit, demonstrating its feasibility for real-time edge and embedded applications.

The remainder of this work is organized into four main sections. The Materials and Methods section presents the dataset and methodology, followed by the Results and Discussion sections that analyze and interpret the findings. Finally, the Conclusion section summarizes the study’s key outcomes.

## 2. Materials and methods

In this section, we have described the datasets and the algorithm used. We have also explained the proposed cascaded model architecture.

### 2.1 Dataset description

Multiple datasets collected from different sources have been utilized in this work to develop and evaluate the proposed framework. Specifically, Dataset-01 is used for the license plate detection (LPD) model, whereas Dataset-02 is utilized for the license plate number recognition (LPNR) model. Table 2 summarizes the dataset statistics, including the total number of images, dataset splits, the number of classes, and the annotation platform.

**Table 2:** Dataset descriptions.

SL	Dataset	Number of Images				Classes	Annotated By
		Total	Train Set	Valid Set	Test Set		
01	Dataset-01	2140	1502	425	213	1	Roboflow
02	Dataset-02	7217	5058	1438	721	104	Roboflow

A total of 9,357 images have been used for model development, while an additional 150 independently collected images have been reserved exclusively for external validation to assess the generalization capability of the proposed framework.

The datasets were compiled from multiple sources, including locally captured photographs, online image crawling, and the Roboflow repository. The locally collected images were acquired using a smartphone camera at several locations in Khulna, Bangladesh, including Gollamari, Sonadanga, and Zero Point. All locally captured images were recorded under daylight and sunny weather conditions. The combined datasets include a diverse range of vehicle categories, such as private cars, buses, trucks, and motorcycles. In addition, Dataset-02 contains numerous cropped license plate images in which the complete vehicle is not visible, as these images are intended solely for character recognition. To ensure a fair evaluation, both Dataset-01 and Dataset-02 were divided into 70% training, 20% validation, and 10% testing sets. The external validation images were not used during model training or hyperparameter tuning and served only to evaluate the robustness of the proposed models on unseen data.

#### 2.1.1 Bangla License Plate Detection dataset.

**Fig 3:**
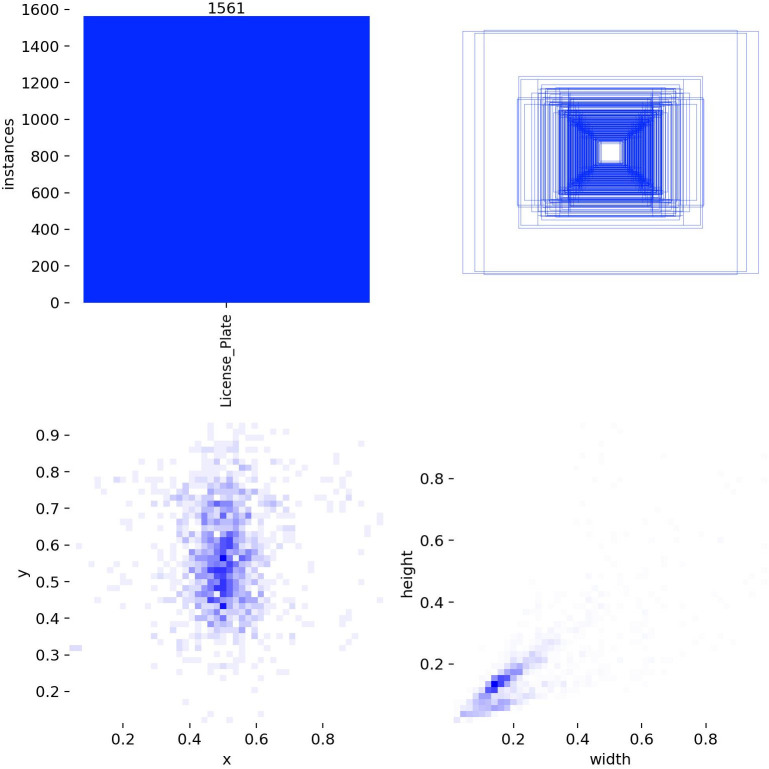
Bounding box distribution and description of Dataset-01.

The Bangla License Plate Detection dataset (Dataset 1), comprising a total of 2,140 images and 2,251 annotated license plate boxes, is utilized to develop a YOLO model for identifying Bangla license plates on vehicles. The visualization in Fig 3 presents the annotation distribution of the Dataset-01. The top-left block indicates that the dataset contains a total of 1,561 annotated instances of the *License_Plate* class, represented as a single solid bar since the model is trained on a single category. The top-right overlay illustrates all normalized bounding boxes plotted together, revealing a concentrated pattern toward the center, indicating that license plates commonly appear in similar regions of the images with relatively consistent aspect ratios.

The bottom-left heatmap displays the distribution of bounding box center coordinates, with dense clustering around x≈0.45−0.55 and y≈0.45−0.65, suggesting that plates typically lie near the central vertical region and the lower half of the image. Finally, the bottom-right heatmap of width versus height shows that most bounding boxes fall within 0.15−0.25 in width and 0.08−0.15 in height, confirming the stable rectangular geometry of license plates across the dataset. Overall, these visual patterns indicate a well-structured and consistent dataset, which supports effective training of the license plate detection model.

#### 2.1.2 License Character Detection dataset.

**Fig 4:**
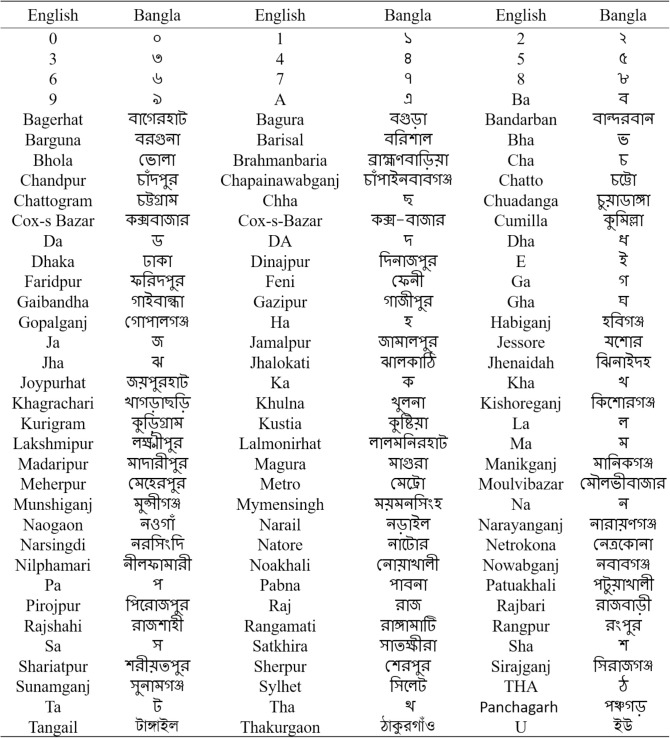
Classes used in the Dataset-02.

Another dataset, the License Character Detection dataset (Dataset-02), which contains 7217 images across 104 classes, as shown in Fig 4, has been used to detect characters within detected license plates. Datasets 01 and 02 have been used to develop the vehicle license plate detection and recognition (VLPDR) model.

**Fig 5:**
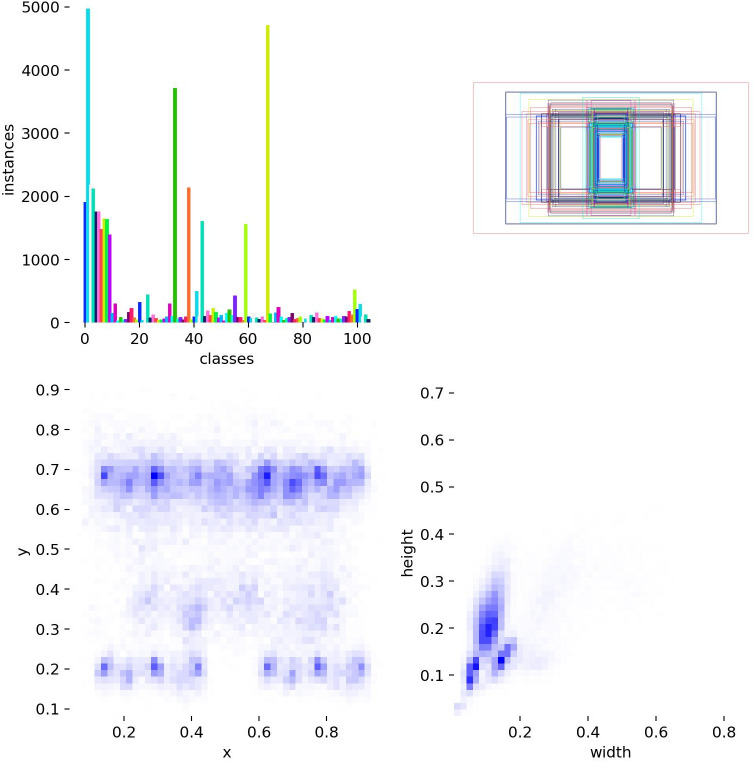
Bounding box distribution and description of the Dataset-02.

To assess the quality and characteristics of the training dataset (Dataset-02) for the proposed license plate character detection model, we analyzed the bounding box distribution statistics generated by the YOLO framework (Fig 5). The class distribution histogram (top-left) reveals a significant imbalance, with a few dominant classes exhibiting high frequency compared to a long tail of underrepresented classes. The bounding box superposition plot (top right) visualizes the aggregate geometry of the annotations, confirming that the ground-truth boxes maintain consistent rectangular aspect ratios centered within the frame. The spatial distribution heatmap (bottom left) indicates a non-random, highly structured arrangement where object centers are clustered in distinct horizontal bands, consistent with the specific capture geometry of the dataset. Finally, the object size analysis (bottom-right) demonstrates that the majority of instances are small-scale relative to the image dimensions, primarily occupying the lower quadrant of width and height, which necessitates a model capable of fine-grained feature extraction for small object detection.

**Fig 6:**
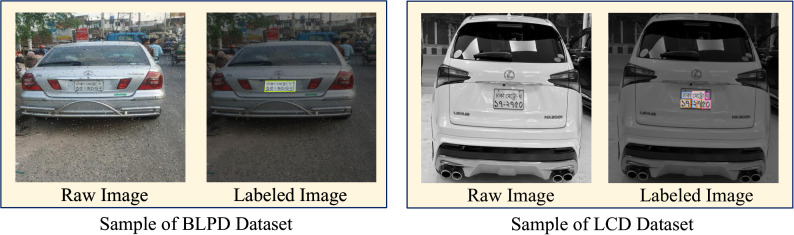
Samples of the datasets: Dataset-01 and Dataset-02.

Table 3 presents the number of classes of the Dataset-02. The class distribution analysis has revealed a noticeable class imbalance within the dataset. The most represented class is Class 1 with 7,396 samples, followed by Metro with 6,292 samples and Dhaka with 4,947 samples. In contrast, several classes contain substantially fewer samples, with Munshiganj having the lowest count of only 2 samples, followed by Nowabganj (11 samples), Bagerhat (37 samples), and Joypurhat and Kustia (45 samples each). Such disparities in class frequencies indicate an imbalanced dataset. Fig 6 shows samples of these datasets: Dataset-01 and Dataset-02.

**Table 3:** Class names and count of Dataset-02.

Class Name	Count	Class Name	Count	Class Name	Count	Class Name	Count	Class Name	Count
0	2778	Chandpur	46	Gopalganj	114	Madaripur	100	Patuakhali	152
1	7396	Chapainawabganj	79	Ha	2179	Magura	65	Pirojpur	120
2	3297	Chatto	603	Habiganj	120	Manikganj	125	Raj	221
3	3110	Chattogram	97	Ja	236	Meherpur	51	Rajbari	101
4	2655	Chha	178	Jamalpur	150	Metro	6292	Rajshahi	88
5	2610	Chuadanga	84	Jessore	289	Moulvibazar	191	Rangamati	73
6	2223	Cox-s Bazar	47	Jha	211	Munshiganj	2	Rangpur	144
7	2431	Cox-s-Bazar	58	Jhalokati	96	Mymensingh	203	Sa	53
8	2411	Cumilla	81	Jhenaidah	162	Na	323	Satkhira	115
9	2071	Da	402	Joypurhat	45	Naogaon	123	Sha	131
A	184	DA	141	Ka	188	Narail	60	Shariatpur	86
Ba	409	Dha	136	Kha	264	Narayanganj	83	Sherpur	85
Bagerhat	37	Dhaka	4947	Khagrachari	151	Narsingdi	101	Sirajganj	139
Bagura	126	Dinajpur	82	Khulna	566	Natore	184	Sunamganj	120
Bandarban	62	E	133	Kishoreganj	117	Netrokona	65	Sylhet	250
Barguna	70	Faridpur	57	Kurigram	113	Nilphamari	84	Ta	713
Barisal	217	Feni	141	Kustia	45	Noakhali	140	Tangail	292
Bha	306	Ga	2853	La	2137	Nowabganj	11	Tha	405
Bhola	105	Gaibandha	54	Lakshmipur	128	Pa	76	THA	179
Brahmanbaria	68	Gazipur	106	Lalmonirhat	102	Pabna	144	Thakurgaon	123
Cha	430	Gha	652	Ma	201	Panchagarh	73	U	188

### 2.2 You Only Look Once (YOLO)

You Only Look Once (YOLO) is a real-time object detection algorithm that frames object detection as a single regression problem, directly predicting bounding box coordinates and class probabilities from full images in one evaluation. Unlike traditional methods such as R-CNN that perform region proposals and classification separately, YOLO processes the image once through a convolutional neural network (CNN), achieving high speed and accuracy simultaneously [8].

**Fig 7:**
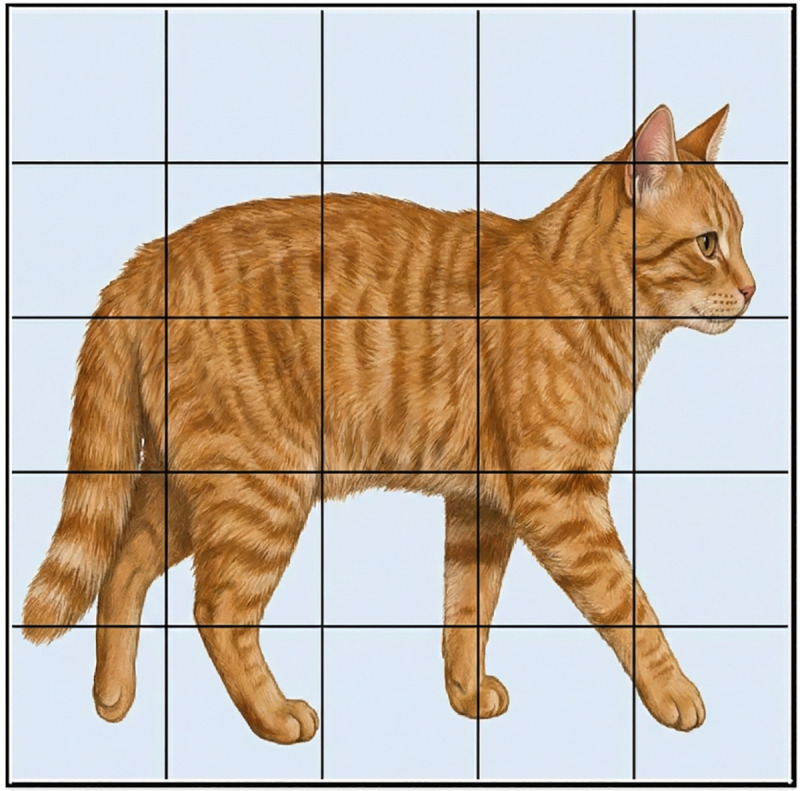
S×S grids of the input image.

1. **Grid-based Detection:** The input image is divided into an S×S grid as shown in Fig 7. Each grid cell is responsible for detecting objects whose centers fall within that cell. For each cell, the model predicts *B* bounding boxes and their confidence scores, along with class probabilities for *C* object classes. The output tensor is of size:

$$\begin{array}{c} S\times S\times (B\times 5+C) \end{array}$$ (1)

where each bounding box prediction contains 5 components: (*x*, *y*, *w*, *h*, *C*) representing the center coordinates (*x*, *y*), width *w*, height *h*, and confidence *C*.

2. **Bounding Box Prediction:** The confidence score *C* reflects the model’s confidence that a bounding box contains an object and how accurate the predicted box is. It is defined as:

$$\begin{array}{c} C=P(\text{object})\times {\text{IOU}}_{\text{pred}}^{\text{truth}} \end{array}$$ (2)

where *P*(object) is the probability that an object exists in the bounding box, and ${\text{IOU}}_{\text{pred}}^{\text{truth}}$ denotes the Intersection over Union between the predicted and ground truth boxes.

The bounding box coordinates are predicted relative to the grid cell:


$$\begin{array}{c} x=\sigma (t_{x})+c_{x},\;y=\sigma (t_{y})+c_{y} \end{array}$$ (3)

$$\begin{array}{c} w=p_{w}e^{t_{w}},\;h=p_{h}e^{t_{h}} \end{array}$$ (4)

where $\left(c_{x},c_{y}\right)$ is the top-left coordinate of the grid cell, $\left(p_{w},p_{h}\right)$ are the anchor box dimensions, and σ denotes the sigmoid function ensuring that *x* and *y* remain within the cell boundaries.

3. **Class Probability Prediction:** For each cell, YOLO predicts conditional class probabilities given that an object exists:

$$\begin{array}{c} P({\text{class}}_{i}|\text{object}) \end{array}$$ (5)

The final class-specific confidence score for each box is obtained as:


$$\begin{array}{c} P({\text{class}}_{i})\times {\text{IOU}}_{\text{pred}}^{\text{truth}}=P({\text{class}}_{i}|\text{object})\times P(\text{object})\times {\text{IOU}}_{\text{pred}}^{\text{truth}} \end{array}$$ (6)

4. **Loss Function:** The YOLO loss function optimizes both localization and classification errors in a unified manner. The total loss is given as:

$$\begin{array}{cc} ℒ= & \;\lambda_{\text{coord}}\sum_{i=0}^{S^{2}}\sum_{j=0}^{B}1_{ij}^{\text{obj}}\left[(x_{i}-{\hat{x}}_{i}{)}^{2}+(y_{i}-{\hat{y}}_{i}{)}^{2}\right]\\ & +\lambda_{\text{coord}}\sum_{i=0}^{S^{2}}\sum_{j=0}^{B}1_{ij}^{\text{obj}}\left[(\sqrt{w_{i}}-\sqrt{{\hat{w}}_{i}}{)}^{2}+(\sqrt{h_{i}}-\sqrt{{\hat{h}}_{i}}{)}^{2}\right]\\ & +\sum_{i=0}^{S^{2}}\sum_{j=0}^{B}1_{ij}^{\text{obj}}(C_{i}-{\hat{C}}_{i}{)}^{2}+\lambda_{\text{noobj}}\sum_{i=0}^{S^{2}}\sum_{j=0}^{B}1_{ij}^{\text{noobj}}(C_{i}-{\hat{C}}_{i}{)}^{2}\\ & +\sum_{i=0}^{S^{2}}1_{i}^{\text{obj}}\sum_{c\in \text{classes}}(p_{i}(c)-{\hat{p}}_{i}(c){)}^{2} \end{array}$$ (7)

where:

- $1_{ij}^{\text{obj}}=1$ if the $j^{th}$ bounding box in cell *i* is responsible for detecting an object, otherwise 0.
- $\lambda_{\text{coord}}$ and $\lambda_{\text{noobj}}$ are hyperparameters controlling the relative importance of coordinate and no-object losses.
- $\left(x_{i},y_{i},w_{i},h_{i},C_{i}\right)$ denote predicted values, and $\left({\hat{x}}_{i},{\hat{y}}_{i},{\hat{w}}_{i},{\hat{h}}_{i},{\hat{C}}_{i}\right)$ denote ground truth values.

**Fig 8:**
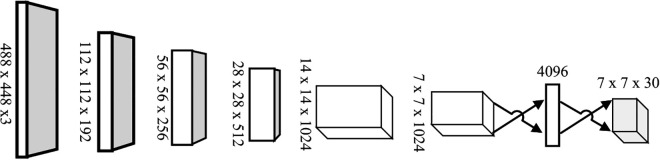
Convolution network structure.

5. **Network Architecture:** The object detection network shown in Fig 8 is a deep convolutional neural network (CNN) consisting of a 24-layer convolutional backbone for feature extraction and two fully connected layers for prediction. The network processes a 448×448×3 input image. The convolutional layers progressively extract features, starting with low-level edges and building to high-level object parts. This stack uses 1×1 convolutional layers to reduce the number of feature channels (a “bottleneck”) and 2×2 max pooling layers to down-sample the spatial dimensions. This process reduces the image from 448×448 to a 7×7×1024 feature map. This feature map is then flattened and passed to the two fully connected layers. The final layer outputs a 7×7×30 tensor. This tensor represents a 7×7 grid laid over the original image, with each cell’s 30 channels encoding predicted bounding box coordinates, object confidence scores, and class probabilities. For training, the 24-layer backbone is first pre-trained on the ImageNet classification dataset (224×224 input). The entire network is then fine-tuned for the detection task at an input resolution of 448×448.

#### 2.2.1 Justification of choosing YOLOv12.

In this work, we have used the base architecture of YOLOv12 as it has successfully integrated an attention-centric design into the YOLO framework, achieving a state-of-the-art latency-accuracy trade-off [29]. Empirical evaluations in the foundational study demonstrate its superiority; for example, YOLOv12-N achieves 40.6% mAP with an inference latency of just 1.64 ms, outperforming YOLOv10-N and YOLOv11-N by 2.1% and 1.2% mAP, respectively [29]. Furthermore, the architecture is highly resource-efficient—variants like YOLOv12-S operate 42% faster than advanced end-to-end detectors like RT-DETRv2-R18, utilizing only 36% of the computation and 45% of the parameters [29]. By utilizing a novel area attention module and Residual Efficient Layer Aggregation Networks (R-ELAN), YOLOv12 provides an optimal foundation for our methodology, allowing us to harness the robust modeling capabilities of attention mechanisms while exceeding the real-time inference efficiency of traditional CNN-based detectors.

### 2.3 Device configuration

To evaluate the performance of the proposed models under varying hardware capabilities, three different computing platforms were used, as detailed in Table 4: a standard laptop without a GPU, a high-performance GPU-enabled desktop, and the Jetson Orin Nano Developer Kit, which serves as the primary target for real-time and real-world deployment. The first platform is a laptop without a dedicated GPU, providing a baseline for CPU-only execution. This device, named Proshen-PC, is equipped with an Intel Core i5-8265U processor (1.60–1.80 GHz) and 8 GB of RAM, running a 64-bit operating system. Its configuration allows us to measure the models’ performance in environments without specialized hardware acceleration.

**Table 4:** Specifications of computing platforms used for testing.

Specification	Laptop (CPU-only)	Desktop (GPU)	Jetson Orin Nano
Processor	Intel Core i5-8265U (1.60–1.80 GHz)	AMD Ryzen 9 7900X (4.70 GHz)	6-core Arm Cortex-A78AE
RAM	8 GB	16 GB	8 GB LPDDR5
GPU	None	NVIDIA GPU (CUDA-enabled)	NVIDIA Ampere (1024 CUDA cores, 32 Tensor cores)
AI Ability	Poor	High	67 INT8 TOPS
Storage	Internal HDD/SSD	Internal HDD/SSD	SD card + external NVMe
OS	64-bit	64-bit	Linux-based embedded OS

**Fig 9:**
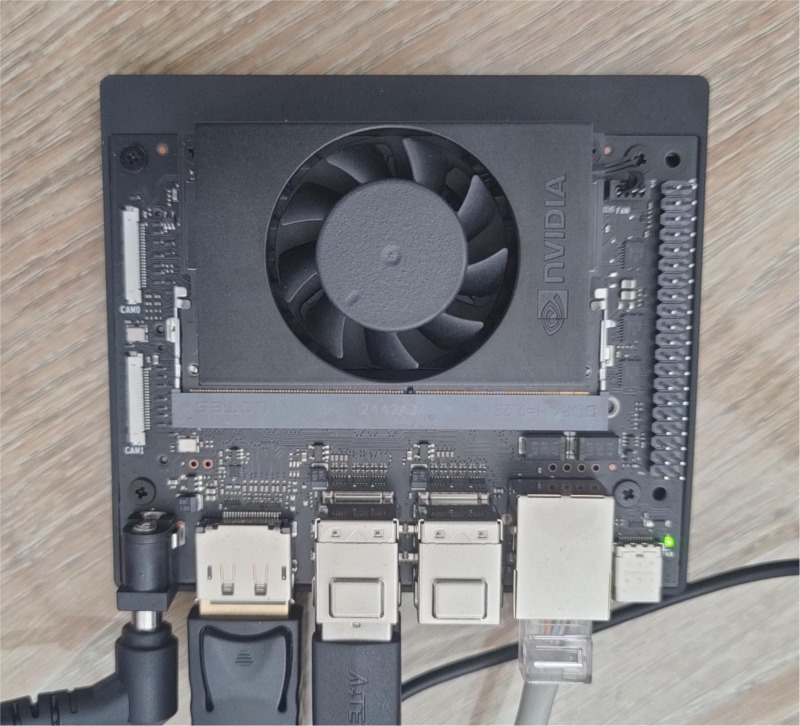
Top view of the used Jetson device.

The second platform is a high-end desktop with GPU capabilities, designed for accelerated inference and training using CUDA-enabled deep learning frameworks. This desktop, identified as DESKTOP-L8ISRDE, features an AMD Ryzen 9 7900X 12-core processor (4.70 GHz) with 16 GB of RAM and a 64-bit operating system. This setup enables the evaluation of models under optimal performance conditions. The third platform is the Jetson Orin Nano Developer Kit shown in Fig 9, a compact edge computing device optimized for AI and real-time computer vision applications. With an NVIDIA Ampere GPU comprising 1024 CUDA cores and 32 tensor cores, a 6-core Arm Cortex-A78AE CPU, 8 GB LPDDR5 memory, and 67 INT8 TOPS AI performance, the Orin Nano provides a specialized architecture for efficient, low-power inference. Its SD card and NVMe support, along with a power range of 7–25 W, make it ideal for deploying the proposed hybrid surveillance models in real-world embedded systems. By testing across these three platforms, we ensured that the proposed models are evaluated both in resource-constrained environments and under conditions optimized for AI performance, demonstrating their adaptability and suitability for real-world deployment.

### 2.4 Proposed VLPDR model architecture

**Fig 10:**
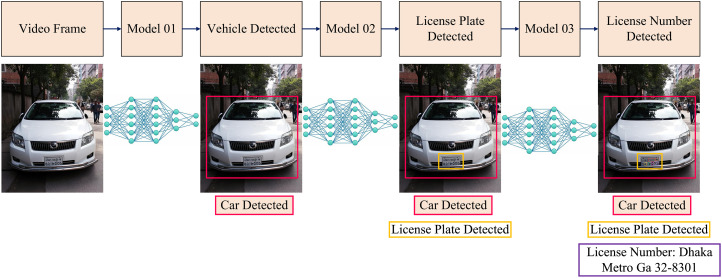
VLPDR model architecture.

The proposed cascaded model for vehicle license plate detection and recognition (VLPDR) model is a cascaded hybrid architecture that integrates three YOLO-based models, each responsible for a dedicated stage of the recognition pipeline, as shown in Fig 10. This multi-stage design has ensured the progressive refinement of information, beginning with vehicle detection, followed by registration plate localization, and ending with the extraction of fine-grained alphanumeric characters. By structuring the process hierarchically, the system has minimized unnecessary computation, reduced false positives, and achieved a higher level of recognition performance in diverse traffic scenes.

In the first stage, a pretrained YOLO model has been employed to detect vehicles appearing in the input frame. Specifically, we have used the Ultralytics YOLO12l model, which was trained on a huge and diverse dataset and therefore provides strong generalization across a wide range of vehicle types and environments [30]. The YOLO12l model contains 26.4 million parameters, enabling it to capture rich spatial and contextual features essential for high-precision vehicle detection [30]. According to the official performance metrics, the model achieves a Mean Average Precision (mAP)${\;}_{50-95}$ of 53.7% at an input resolution of 640 pixels, with an inference speed of 6.77 ms on an NVIDIA T4 TensorRT environment and a computational complexity of 88.9 GFLOPs. When compared to YOLO11l, YOLO12l demonstrates a performance improvement of +0.4% mAP while maintaining competitive inference speed (–8%). These characteristics make YOLOv12 a suitable choice for the proposed pipeline, as it ensures robust detection of common vehicle classes, including cars, buses, trucks, and motorcycles. The bounding box produced in this stage provides the initial region of interest (ROI) for subsequent processing. Since this model focuses exclusively on vehicle identification, its output has served as the foundational input for the next stages of the VLPDR architecture.

In the second stage, the vehicle region detected in the first stage is passed through a custom-trained YOLO model named the License Plate Detection (LPD) model, which has been developed to detect Bangladeshi vehicle registration plates. By restricting the search space to the detected vehicle region of interest (ROI), this model has achieved improved plate localization accuracy, even in cluttered or dynamic environments. The bounding box corresponding to the registration plate has then been mapped back to the original frame to ensure precise extraction of the plate area. For training this model, we have utilized the Bangla License Plate Detection dataset, which consists of a total of 2,140 images and 2,251 annotated license plate boxes. This dataset has provided diverse vehicle and license plate scenarios, enabling the model to generalize effectively in real-world traffic conditions. For model development, the LPD model was trained using the Ultralytics YOLO framework in a Google Colab environment equipped with NVIDIA GPUs such as the Tesla T4 (16 GB), which is commonly provided by the platform for deep learning tasks. The training process has used 100 epochs with an input image size of 640×640 pixels. External validation has been conducted on two platforms: a Computer and Jetson Orin.

**Algorithm 1. VLPDR model algorithm.**

**Require:** Input frame *I*, confidence threshold τ

**Ensure:** Detected vehicle class, plate location, and reconstructed registration number

1: Load pretrained YOLO model *M*_1_ (Vehicle detection model)

2: Load custom YOLO model *M*_2_ (LPD model)

3: Load custom YOLO model *M*_3_ (LPNR model)

4: **for** each frame *I* **do**

5:  $V\leftarrow M_{1}(I)$ ▷ Detect vehicles

6:  **for** each detection $v_{i}\in V$ **do**

7:   **if** $score(v_{i})<\tau$ **then**

8:    **continue**

9:   **end if**

10:   Extract vehicle region $R_{i}=I[b_{i}]$

11:   $P\leftarrow M_{2}(R_{i})$ ▷ Detect license plate

12:   **for** each plate $p_{j}\in P$ **do**

13:    **if** $score(p_{j})<\tau$ **then**

14:     **continue**

15:    **end if**

16:    Convert $p_{j}$ to absolute coordinates

17:    Extract plate region $R_{i}^{p}=I[p_{j}^{abs}]$

18:    $C\leftarrow M_{3}(R_{i}^{p})$ ▷ Detect characters

19:    Sort *C* by left-to-right bounding box order

20:    Construct registration string $L=\underset{c_{k}\in C}{⨁}label(c_{k})$

21:    Display vehicle box, plate box, and license number *L*

22:   **end for**

23:  **end for**

24: **end for**

In the third stage, the extracted registration plate is processed using a custom YOLO model named the LPNR model, for character-level detection. This model was trained on Dataset-02, which contains the Bangla alphabet and digits commonly appearing on Bangladeshi number plates, comprising a total of 7217 images, 104 classes, and 63,879 annotated boxes for the alphanumeric values of the plates. This dataset has provided diverse vehicle and license plate scenarios, enabling the model to generalize effectively in real-world traffic conditions. For model development, the LPNR model was trained using the same GPU and environment. The training process was configured for 70 epochs with an input image size of 640×640 pixels. External validation has been conducted on two platforms: a Computer and a Jetson Orin.

The output has consisted of individual character bounding boxes, each labeled with its corresponding alphanumeric class. To reconstruct the registration number, the detected characters have been organized as City-Metro (if available)-Character-Number, for example, Dhaka Metro Ga 328301. The cascaded design of these three models has enabled the VLPDR system to operate accurately while ensuring high robustness and accuracy. The hierarchical flow—from vehicle detection to plate localization to character recognition—has ensured that each stage benefits from the contextual constraints imposed by the previous one, resulting in fewer misclassifications and improved generalization under varying lighting, occlusion, and camera conditions.

The VLPDR cascaded algorithm, as shown in Algorithm 1, has been designed as a cascaded three-stage pipeline for vehicle license plate and character detection. Initially, each video frame is processed using a pretrained YOLO model (*M*_1_) to detect vehicles, where only detections above a specified confidence threshold (τ) are considered. For each detected vehicle, the corresponding region of interest (ROI) is extracted and passed to a custom-trained YOLO model (*M*_2_) to localize the registration plate within the vehicle. Plates detected with sufficient confidence are then mapped back to the original frame to obtain absolute coordinates, and the plate region is cropped. This cropped plate is subsequently analyzed using a second custom YOLO model (*M*_3_) to detect and classify individual alphanumeric characters. The detected characters are sorted from left to right based on their bounding box positions and concatenated to reconstruct the full license number. Finally, the algorithm overlays the vehicle and plate bounding boxes along with the reconstructed license number on the video frame, providing an end-to-end hierarchical detection and recognition system that efficiently integrates vehicle, plate, and character-level information.

### 2.5 Evaluation metrics

In this work, the performance of the proposed model has been evaluated using standard object detection metrics, as well as computational complexity across different platforms. The primary metrics considered are:

- **Accuracy:** Measures the proportion of correctly detected objects among all detections:

$$\begin{array}{c} \text{Accuracy}=\frac{TP+TN}{TP+TN+FP+FN} \end{array}$$ (8)

where *TP*, *TN*, *FP*, and *FN* denote true positives, true negatives, false positives, and false negatives, respectively.

- **Precision and Recall of Bounding Boxes:** These metrics evaluate the quality of predicted bounding boxes:

$$\begin{array}{c} \text{Precision}=\frac{TP}{TP+FP},\;\text{Recall}=\frac{TP}{TP+FN} \end{array}$$ (9)

- **F1-score:** The harmonic mean of precision and recall, which balances both metrics:

$$\begin{array}{c} F1=2\times \frac{\text{Precision}\times \text{Recall}}{\text{Precision}+\text{Recall}} \end{array}$$ (10)

- **Mean Average Precision (mAP):** The model has been evaluated using mean Average Precision at different Intersection over Union (IoU) thresholds: - mAP@50: Average precision at IoU = 0.5 - mAP@50–95: Average precision across IoU thresholds from 0.5 to 0.95

$$\begin{array}{c} \text{mAP}=\frac{1}{N}\sum_{i=1}^{N}AP_{i} \end{array}$$ (11)

where $AP_{i}$ is the average precision for the *i*-th class, and *N* is the total number of classes.

To evaluate the similarity between ground-truth and predicted license plate numbers, we have used the *Levenshtein similarity* [31], which measures the minimum number of insertions, deletions, and substitutions required to transform one string into another. The normalized score ranges from 0 to 1, where 1 indicates an exact match, effectively capturing minor recognition errors in automatic license plate recognition.

- **Execution Time:** The computational complexity of the model has been analyzed both during training (epoch-wise) and testing (per image) on different hardware platforms:
  1. **Laptop without GPU:** Intel^®^ Core™ i5-8265U CPU @ 1.60 GHz (1.80 GHz) with 8 GB RAM (7.89 GB usable); CPU-only inference.
  2. **Desktop GPU-enabled computer:** AMD Ryzen 9 7900X processor with 16 GB RAM and NVIDIA GeForce RTX 3050 GPU; GPU-based inference has been used.
  3. **Jetson Orin Nano:** 6-core Arm^®^ Cortex-A78AE v8.2 64-bit CPU with 1.5 MB L2 + 4 MB L3 cache; CPU-only inference.

## 3. Results

The vehicle license plate detection and recognition (VLPDR) model was developed using three integrated components: two custom-built models—one for license plate detection and another for character extraction from the plate—designed specifically for this research, and one pretrained model (YOLO12l) from Ultralytics for vehicle detection. The License Plate Detection (LPD) model has utilized Dataset-01, which has 2140 images and a single class. The Dataset-02, comprising 7217 pictures and 104 classes, has been used in the LPNR model. Table 5 presents the experimental setting for reproducibility.

**Table 5:** Training configurations of the LPD and LPNR models.

Parameter	LPD Model	LPNR Model
Architecture	YOLOv12	YOLOv12
Input Image Size	640×640	640×640
Batch Size	16	16
Number of Epochs	100	70
Optimizer	Auto	Auto
Initial Learning Rate (*lr*_0_)	0.01	0.01
IoU/NMS Threshold	0.7	0.7
Weight Decay	0.0005	0.0005
Data Augmentation	No	No
Cosine Learning Rate Scheduling	False	False
Deterministic Training	True	True
Dropout	0.0	0.0
Dataset Fraction Used	1.0	1.0
Training Mode	Train	Train
Early Stopping	Not Applied	Not Applied
Patience	100	100

### 3.1 Result of License Plate Detection (LPD) model

During the License Plate Detection (LPD) model training, we have monitored various performance metrics to assess the quality and stability of the learning process. These metrics include the number of epochs, training time, training losses (box loss, classification loss, and distribution focal loss), and validation losses. In addition, evaluation metrics such as box precision, recall, and mean Average Precision at Intersection over Union (IoU) 0.5 (mAP@50), and mean Average Precision across Intersection over Union thresholds from 0.5 to 0.95 (mAP@50–95) have been considered.

#### 3.1.1 Training and validation performances.

**Fig 11:**
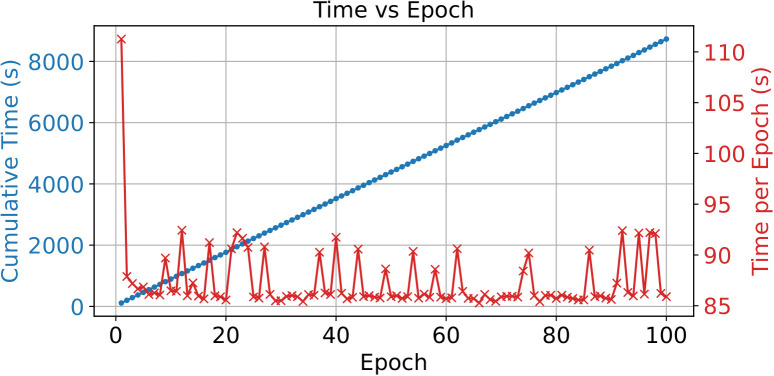
LPD model train time vs epoch.

**Fig 12:**
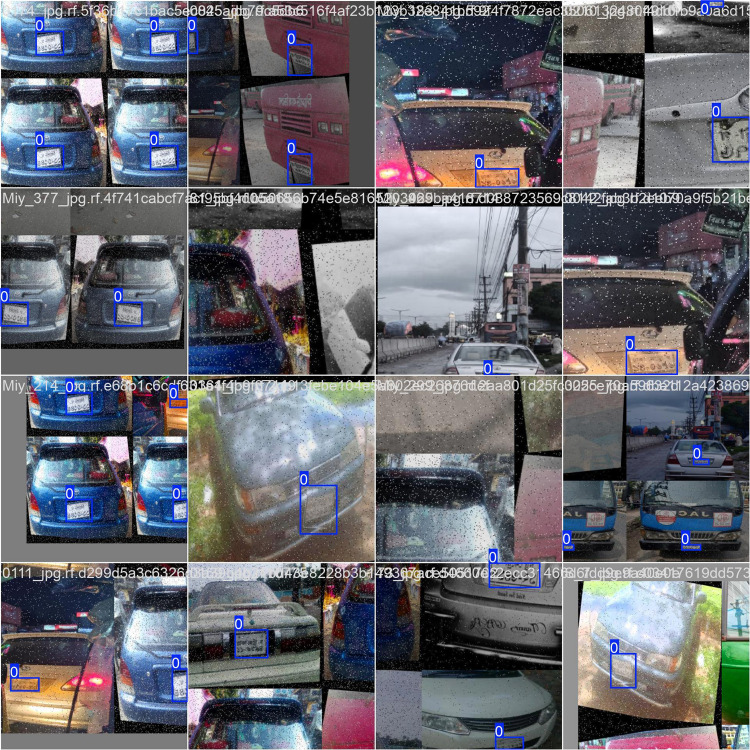
A LPD model training batch.

**Fig 13:**
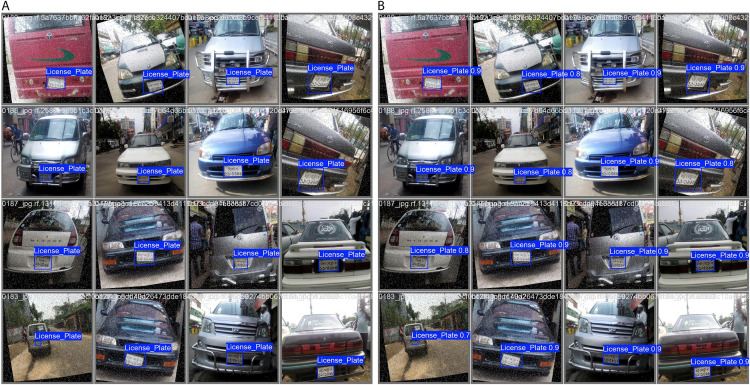
Comparison of LPD model validating batch: ground truth vs predictions. (a) Validating batch with labels. (b) Validating batch with predictions.

Fig 11 illustrates a highly stable training process for the LPD model over 100 epochs. The Cumulative Time (blue line) shows a perfectly linear increase, reaching approximately 8730.75 seconds (about 2.42 hours), which indicates that the computational cost remained consistent throughout the training duration. The Time per Epoch (red line) displays a characteristic spike during the first epoch (>110 seconds)—likely caused by initial overhead such as memory allocation, caching, or CUDA kernel compilation—before stabilizing significantly to an average 87.3 seconds per epoch, with only minor fluctuations attributable to normal system variations or data loading jitter. Figs 12, 13a and 13b have illustrated a single batch that has been used during training, validation with ground-truth labels, and validation with the corresponding predicted outputs.

**Fig 14:**
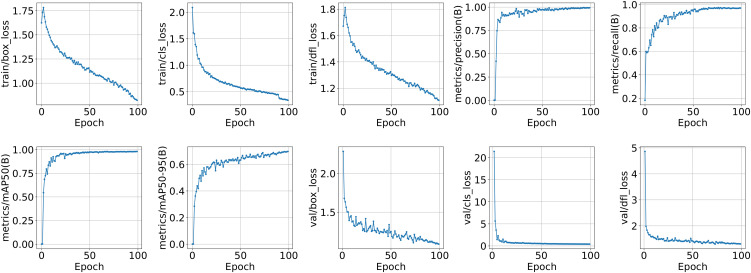
LPD model performances across the epochs during training.

Fig 14 presents a quantitative analysis of the model’s training performance over 100 epochs, including convergence of the loss functions and improvements in detection metrics. The training process exhibited a consistent downward trajectory in loss values, signifying effective parameter optimization. Specifically, the training bounding box loss (train/box_loss) decreased substantially from an initial value of 1.624 to 0.823, while the classification loss (train/cls_loss) dropped from 2.089 to 0.338. Similarly, the distribution focal loss (train/dfl_loss), which refines localization precision, fell from 1.672 to 1.109. This learning progress was mirrored in the validation phase, where the losses demonstrated strong generalization capabilities; by the final epoch, the validation box loss (val/box_loss), classification loss (val/cls_loss), and distribution focal loss (val/dfl_loss) had converged to 1.086, 0.412, and 1.305, respectively, indicating minimal overfitting.

In terms of detection accuracy, the model achieved significant improvements across all performance metrics. The precision (metrics/precision(B)) surged from a baseline of 0.001 to a final value of 0.993, reflecting a high resistance to false positives. Concurrently, the recall (metrics/recall(B)) increased from 0.183 to 0.970, demonstrating the model’s proficiency in retrieving the majority of ground truth instances. The mean average precision scores further corroborated the overall detection capability: the mAP at 0.5 IoU threshold (metrics/mAP50(B)) reached a remarkable 0.981. In contrast, the more stringent mAP averaged over 0.5–0.95 IoU thresholds (metrics/mAP50-95(B)) achieved 0.697. These results collectively confirm that the model delivers robust performance with high localization accuracy and classification reliability.

**Fig 15:**
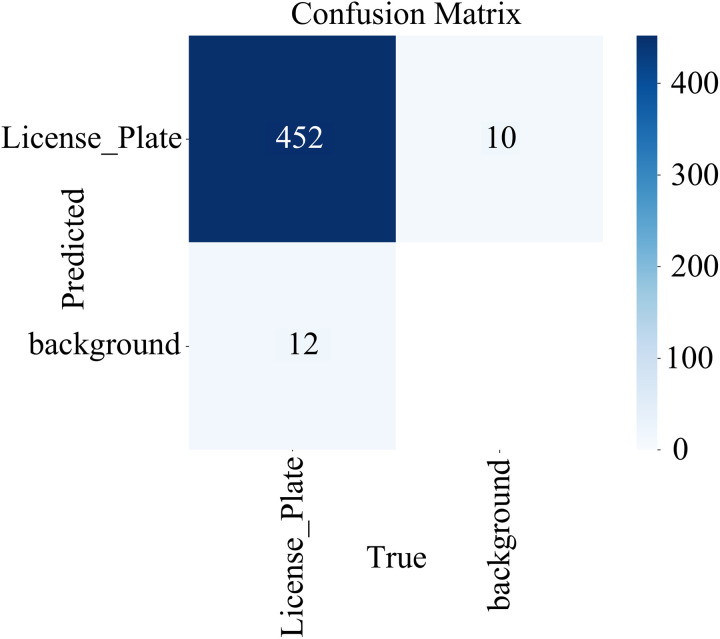
LPD model validation confusion matrix.

Table 6 shows the validation performances of the LPD model on 425 images with 464 instances. The LPD model has achieved up to 99.3% precision, 97% recall, and 97.4% mAP@50. The confusion matrix, shown in Fig 15, provides an analysis of the single-class License Plate detection task, highlighting specific misclassification patterns involving the background. The model exhibited 12 instances of False Negatives (FN), where actual license plates were incorrectly classified as background and not detected. Conversely, the model produced 10 False Positives (FP), where background regions were erroneously predicted as license plates, thereby reducing the precision of the detection.

**Table 6:** LPD model validation results.

Metric	Precision (P)	Recall (R)	mAP@50	mAP@50–95
Value	0.993	0.97	0.974	0.674

**Fig 16:**
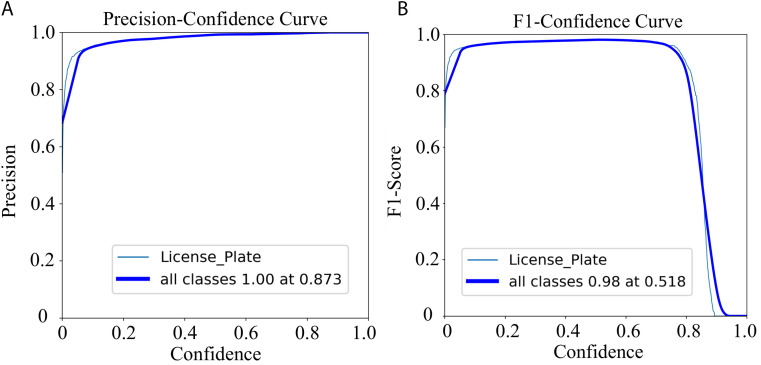
LPD model validation confidence curves. (a) Precision confidence curve. (b) F1 confidence curve.

The performance of the license plate detection model is quantitatively assessed through the Precision-Confidence (Fig 16a) and F1-Confidence curves (Fig 16b), illustrating the model’s reliability across varying thresholds. The Precision-Confidence curve demonstrates a strong positive correlation, where the model achieves perfect precision (1.00) at a confidence threshold of 0.873, indicating that high-confidence predictions are free of false positives. Furthermore, the F1-Confidence curve, which represents the harmonic mean of precision and recall, identifies the optimal operating point for the detector; the model attains a peak F1 score of 0.98 at a confidence threshold of 0.518.

#### 3.1.2 Test performances.

**Fig 17:**
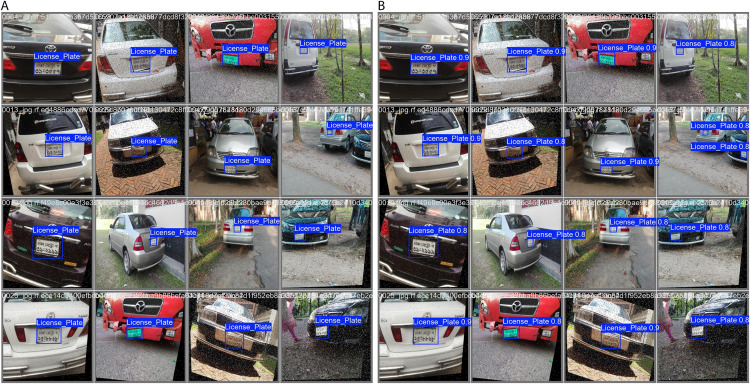
Comparison of LPD model testing batch: ground truth vs predictions. (a) Testing batch with labels. (b) Testing batch with predictions.

The evaluation of the LPD model on the test set demonstrates robust detection capabilities across varied conditions. Fig 17a and 17b display a representative batch of 16 test images, comparing the ground-truth labels against the model’s predictions. The visual results confirm that the model successfully localizes license plates even in challenging scenarios involving occlusion, rotation, and noise.

The LPD model has been evaluated on the test set containing 213 images and 226 annotated license plate instances. The results in Table 7 show that the model has achieved a precision of 0.986 and a recall of 0.965, indicating accurate detection of license plate regions. The mAP@50 value of 0.963 reflects strong localization performance at the standard IoU threshold, whereas the mAP@50–95 score of 0.685 reflects performance across more restrictive IoU thresholds.

**Table 7:** LPD model test results.

Metric	Precision (P)	Recall (R)	mAP@50	mAP@50–95
Value	0.986	0.965	0.963	0.685

**Fig 18:**
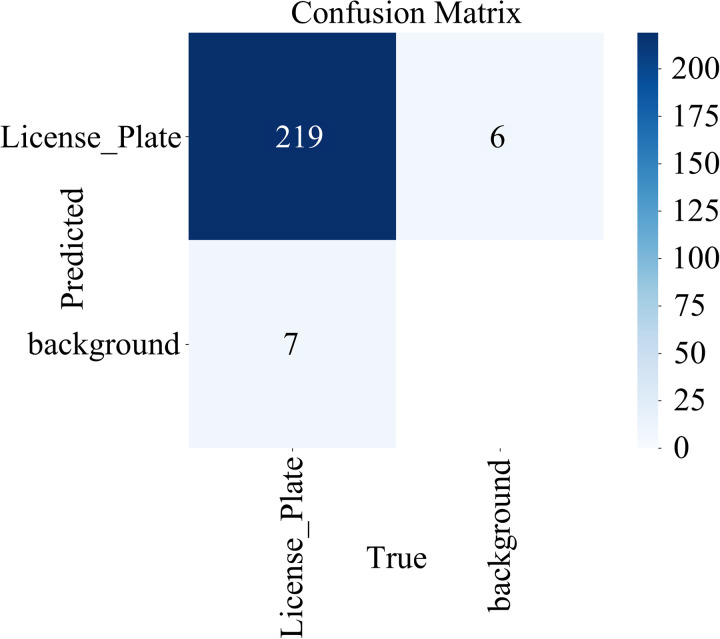
LPD model testing confusion matrix.

**Fig 19:**
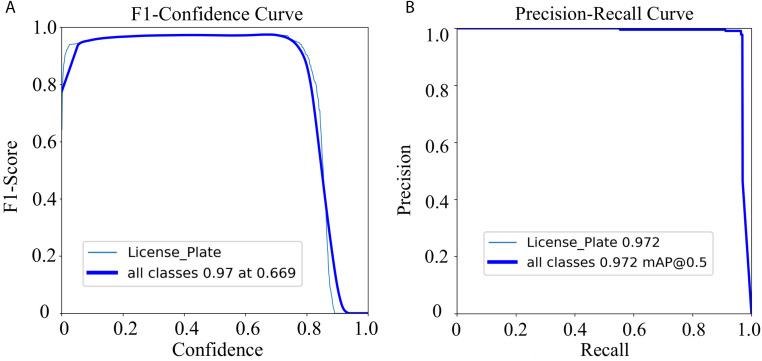
F1 confidence curve of the LPD model test set.

This qualitative assessment is quantitatively supported by the confusion matrix in Fig 18, which reveals a high true positive rate with 219 correctly identified license plates and minimal misclassifications (6 background errors and 7 missed detections), indicating a strong ability to distinguish between the object of interest and the background. Furthermore, Fig 19 illustrates the trade-off between precision and recall, showing that the model maintains a high F1 score of 0.97 at a confidence threshold of 0.669. This optimal operating point suggests that the model is highly reliable, striking a balance that minimizes false positives while maximizing true license plate detection.

### 3.2 Result of License Plate Number Recognition (LPNR) model

In this subsection, the performance of the LPNR model is demonstrated, including training, validation, and testing results.

#### 3.2.1 Training and validation performances.

**Fig 20:**
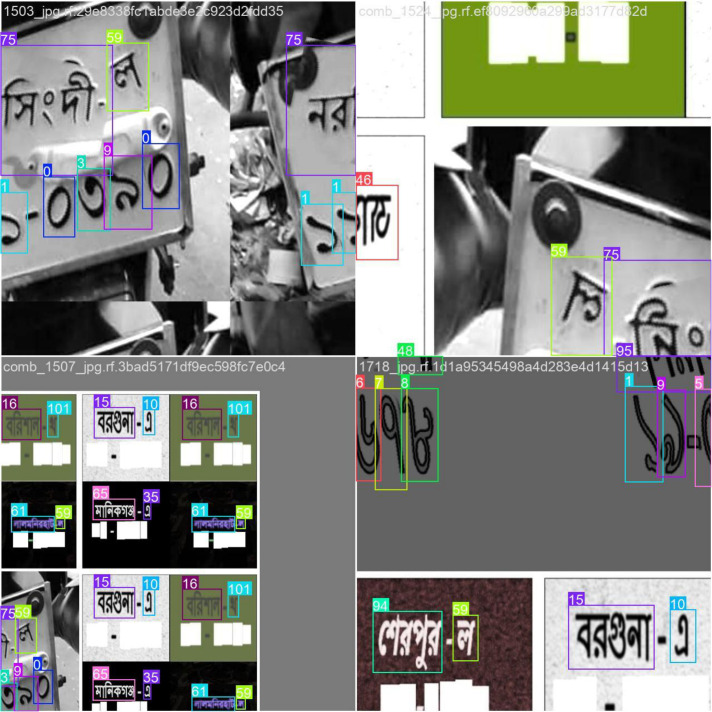
A LPNR model training batch.

To qualitatively validate the learning efficacy of the LPNR model framework, we examined the visual outputs generated during the training and validation phases. The training batch samples (Fig 20) illustrate the dataset’s diversity, featuring varied illumination conditions, noise levels, and perspective distortions inherent in real-world capture.

**Fig 21:**
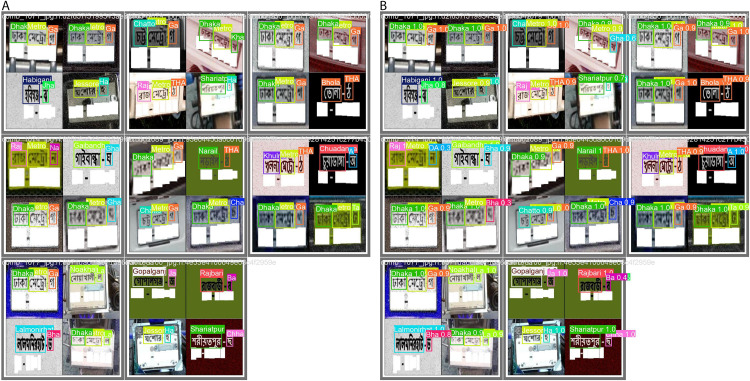
LPNR model validation batch: true labels and corresponding predictions. (a) Validating batch with labels. (b) Validating batch with predictions.

To assess inference performance, we compared the ground-truth annotations (Fig 21a) with the model’s predictions (Fig 21b) on the validation set. The visual comparison demonstrates precise localization accuracy, with the predicted bounding boxes aligning tightly with the ground truth across diverse classes, including district names (e.g., ‘Dhaka’, ‘Jessore’) and class identifiers. Furthermore, the model exhibits high confidence scores, predominantly exceeding 0.9, confirming its robustness in distinguishing and classifying individual Bengali characters within the structured layout of license plates.

**Fig 22:**
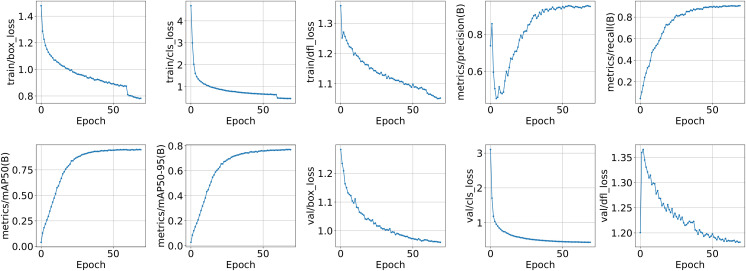
LPNR model performances across the epochs during training.

The performance evolution of the LPNR model over 70 epochs, as illustrated in Fig 22, demonstrates rapid convergence and high detection accuracy. The loss curves exhibit a sharp downward trajectory, with the classification loss notably dropping from an initial high of ~4.69 to a stable ~0.46, and box regression loss reducing by approximately 50%, indicating effective optimization of both class separability and localization. In terms of detection capability, the precision and recall metrics show a strong correlation, peaking at 0.955 and 0.906, respectively. This balanced performance culminates in a Mean Average Precision (mAP) of 95.0% at IoU 0.5, while the more rigorous mAP@0.5:0.95 metric reaches 77.1%, validating the model’s robustness in accurately detecting and localizing characters even under strict intersection constraints.

**Fig 23:**
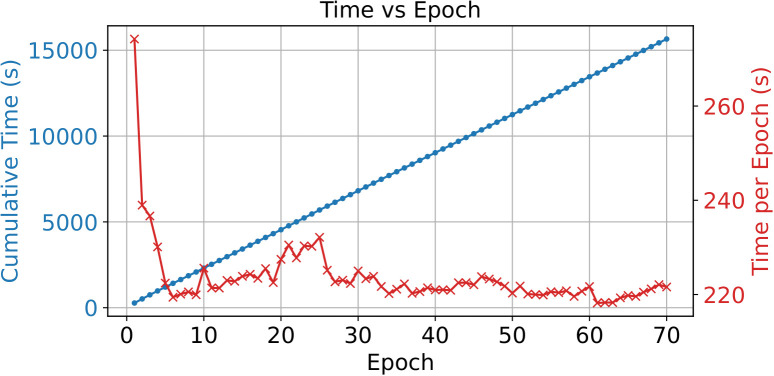
LPNR model train time vs epoch.

The training profile for the LPNR model, spanning over 70 epochs, shown in Fig 23, reveals a total duration of 15,654.2 seconds (approximately 4.35 hours), with a consistent linear increase in Cumulative Time (blue line). The Time per Epoch (red line) follows a typical “warm-up” pattern, starting with a significant spike (likely due to initialization overhead) before dropping sharply. Unlike the previous model, this run exhibits a noticeable period of instability between epochs 15 and 25, where the time fluctuates upwards, potentially indicating temporary resource contention or variable data loading speeds. However, beyond epoch 30, the training stabilizes effectively, adhering closely to an average of 223.63 seconds, suggesting the system recovered from the early variances to maintain a steady computational throughput.

**Fig 24:**
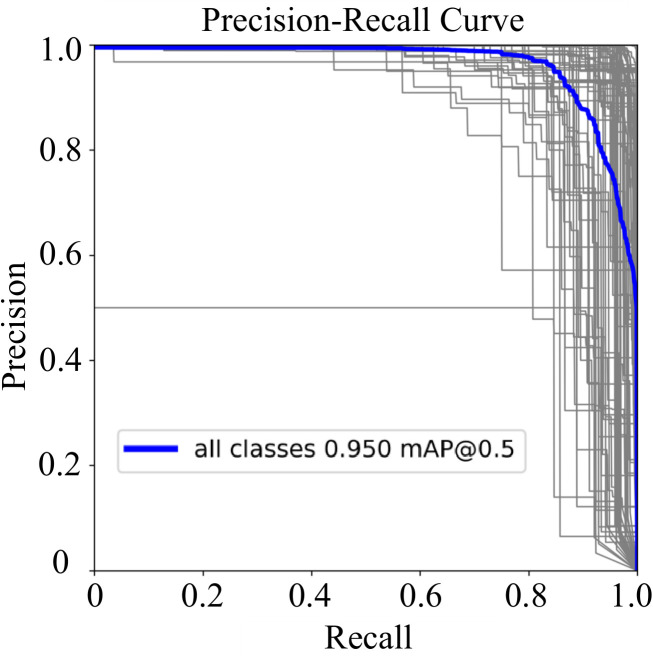
LPNR model validation Precision–Recall curve.

**Fig 25:**
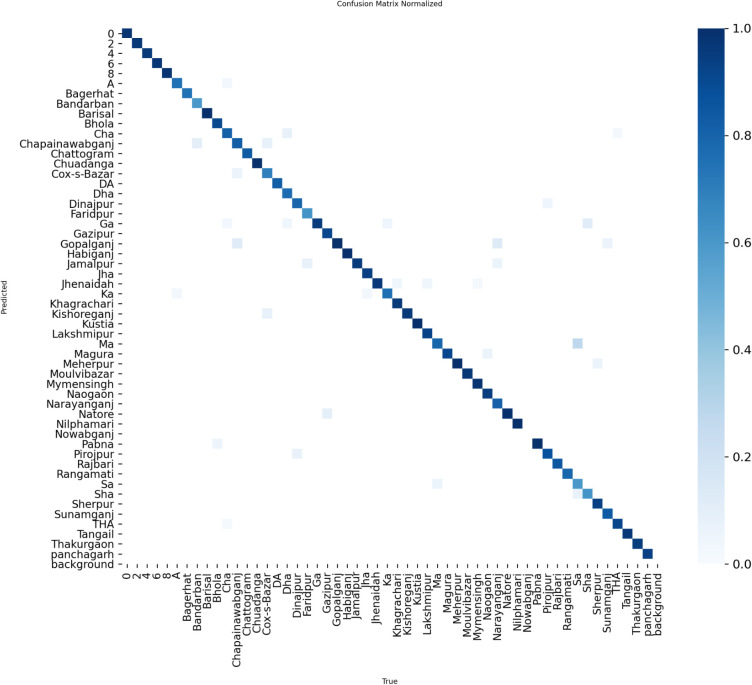
Valid set normalized confusion matrix of LPNR model.

The LPNR model has achieved strong detection performance for all classes on the validation dataset, as indicated by the bounding box evaluation metrics as presented in Table 8. The model has obtained a precision of 0.952, demonstrating a high capability to correctly identify license plates with minimal false positives. A recall value of 0.905 indicates that the majority of actual license plates have been successfully detected by the model. Furthermore, the model has reached an mAP@50 of 0.95 (presented in Fig 24), reflecting excellent detection accuracy at an Intersection over Union (IoU) threshold of 0.5, while the mAP@50–95 score of 0.77 indicates consistent performance across multiple IoU thresholds, confirming the robustness and reliability of the bounding box predictions. The normalized confusion matrix in Fig 25 exhibits strong diagonal dominance, reflecting minimal inter-class confusion and confirming the model’s capability to distinguish visually similar Bengali characters and district identifiers with high accuracy.

**Table 8:** LPNR model validation results.

Metric	Precision (P)	Recall (R)	mAP@50	mAP@50–95
Value	0.952	0.905	0.95	0.77

#### 3.2.2 Test performances.

The proposed Bangladeshi License Plate Character Detection model demonstrates exceptional performance across multiple evaluation metrics. As evidenced by the comprehensive results, the model achieved an overall mean Average Precision (mAP50) of 95% and mAP50-95 of 76.7%, indicating robust detection capabilities across various IoU thresholds shown in Table 9. With a precision of 95% and a recall of 89.8%, the model strikes an optimal balance between accurate detections and comprehensive coverage of all license plate characters. The evaluation was conducted on a substantial dataset comprising 721 images, which contained 6,292 individual character instances, representing diverse Bangladeshi license plate formats across multiple districts. Notably, the model exhibited outstanding performance on major metropolitan areas, with Dhaka achieving mAP50-95 of 0.864 and Metro regions reaching 0.887, demonstrating the model’s effectiveness in urban environments with complex traffic conditions.

**Table 9:** Complete performances of the LPNR model on the test set (continued).

SL.	Class	Images	Instances	Precision	Recall	mAP50	mAP50-95
1	0	234	280	0.996	0.939	0.985	0.663
2	1	448	781	0.999	0.956	0.993	0.667
3	2	292	365	0.99	0.964	0.992	0.665
4	3	292	350	1	0.966	0.991	0.693
5	4	248	278	1	0.961	0.988	0.665
6	5	254	280	0.989	0.948	0.988	0.664
7	6	221	245	0.992	0.976	0.992	0.708
8	7	245	266	0.999	0.966	0.99	0.665
9	8	226	249	0.996	0.927	0.989	0.687
10	9	207	232	0.995	0.936	0.989	0.69
11	A	8	9	0.867	0.667	0.775	0.526
12	Ba	42	46	0.923	0.891	0.949	0.759
13	Bagerhat	5	5	0.992	0.8	0.92	0.855
14	Bagura	17	17	0.972	0.941	0.99	0.765
15	Bandarban	6	6	0.954	1	0.995	0.918
16	Barguna	6	6	0.936	0.667	0.927	0.773
17	Barisal	19	20	0.987	0.95	0.951	0.814
18	Bha	24	25	0.95	0.768	0.895	0.68
19	Bhola	10	10	0.969	1	0.995	0.813
20	Brahmanbaria	7	7	1	0.918	0.995	0.828
21	Cha	38	40	0.97	0.811	0.854	0.612
22	Chandpur	7	7	0.962	1	0.995	0.794
23	Chapainawabganj	3	3	0.593	0.667	0.789	0.677
24	Chatto	50	53	1	0.943	0.95	0.75
25	Chattogram	6	6	0.936	0.667	0.74	0.668
26	Chha	16	16	0.923	0.754	0.814	0.598
27	Chuadanga	6	6	1	0.941	0.995	0.879
28	Cox-s Bazar	5	5	0.941	1	0.995	0.925
29	Cox-s-Bazar	6	6	0.886	0.833	0.931	0.717
30	Cumilla	10	10	0.992	1	0.995	0.856
31	DA	20	20	1	0.862	0.995	0.658
32	Da	29	30	0.913	0.8	0.906	0.726
33	Dha	9	9	1	0.839	0.984	0.739
34	Dhaka	298	446	0.996	0.962	0.994	0.864
35	Dinajpur	10	10	0.949	0.7	0.7	0.603
36	E	13	13	0.955	0.769	0.907	0.573
37	Faridpur	5	5	0.962	1	0.995	0.85
38	Feni	15	15	0.975	1	0.995	0.8
39	Ga	205	265	0.969	0.929	0.977	0.775
40	Gaibandha	6	6	0.933	0.833	0.835	0.771
41	Gazipur	5	5	0.946	1	0.995	0.785
42	Gha	45	47	0.952	0.915	0.938	0.747
43	Gopalganj	5	5	0.887	1	0.995	0.774
44	Ha	171	195	0.993	0.959	0.986	0.705
45	Habiganj	14	14	1	0.974	0.995	0.802
46	Ja	17	18	0.835	0.722	0.808	0.674
47	Jamalpur	15	15	0.966	1	0.995	0.892
48	Jessore	24	24	0.959	1	0.995	0.8
49	Jha	18	18	0.997	0.889	0.987	0.754
50	Jhalokati	9	9	0.945	1	0.995	0.832
51	Jhenaidah	15	16	0.96	0.938	0.968	0.808
52	Joypurhat	2	2	0.757	0.5	0.638	0.42
53	Ka	17	17	1	0.867	0.995	0.781
54	Kha	21	21	0.937	0.712	0.887	0.733
55	Khagrachari	11	11	0.908	0.901	0.909	0.783
56	Khulna	44	45	0.95	0.956	0.968	0.819
57	Kishoreganj	7	7	1	0.981	0.995	0.821
58	Kurigram	11	11	1	0.904	0.915	0.748
59	Kustia	6	6	0.888	1	0.995	0.863
60	La	204	220	0.99	0.95	0.988	0.69
61	Lakshmipur	11	11	0.966	0.909	0.934	0.832
62	Lalmonirhat	11	11	0.945	0.818	0.98	0.853
63	Ma	15	15	1	0.815	0.995	0.755
64	Madaripur	8	8	0.929	1	0.995	0.978
65	Magura	7	7	0.868	0.948	0.943	0.816
66	Manikganj	14	14	0.965	0.929	0.99	0.836
67	Meherpur	9	9	0.921	1	0.995	0.957
68	Metro	356	554	0.995	0.989	0.995	0.887
69	Moulvibazar	17	17	0.971	0.941	0.945	0.802
70	Mymensingh	8	8	0.969	1	0.995	0.857
71	Na	30	31	0.964	0.874	0.96	0.615
72	Naogaon	14	14	0.973	1	0.995	0.72
73	Narail	5	5	0.959	1	0.995	0.915
74	Narayanganj	2	2	0.805	1	0.995	0.696
75	Narsingdi	6	6	1	0.878	0.995	0.927
76	Natore	12	12	0.968	0.917	0.942	0.821
77	Netrokona	5	5	0.93	1	0.995	0.74
78	Nilphamari	6	6	0.868	1	0.995	0.776
79	Noakhali	19	19	0.943	1	0.995	0.841
80	Nowabganj	3	3	1	0	0.995	0.929
81	Pa	7	7	1	0.857	0.864	0.6
82	Pabna	14	14	0.978	1	0.995	0.844
83	Patuakhali	11	11	0.966	0.909	0.919	0.812
84	Pirojpur	14	14	1	0.975	0.995	0.902
85	Raj	19	19	0.971	0.947	0.988	0.812
86	Rajbari	11	12	0.896	1	0.995	0.894
87	Rajshahi	16	16	0.958	0.938	0.935	0.814
88	Rangamati	10	10	0.896	0.866	0.891	0.683
89	Rangpur	21	21	1	0.905	0.92	0.723
90	Sa	2	2	0.429	0.5	0.495	0.346
91	Satkhira	11	12	1	0.942	0.995	0.839
92	Sha	4	4	0.893	0.75	0.945	0.87
93	Shariatpur	5	5	1	0.751	0.92	0.835
94	Sherpur	13	13	0.928	0.99	0.99	0.824
95	Sirajganj	14	14	0.953	1	0.995	0.819
96	Sunamganj	14	15	0.964	0.867	0.929	0.801
97	Sylhet	18	18	0.991	1	0.995	0.812
98	THA	14	14	0.973	0.929	0.925	0.657
99	Ta	67	68	0.967	0.926	0.988	0.733
100	Tangail	17	17	0.972	1	0.995	0.877
101	Tha	32	32	1	0.827	0.925	0.616
102	Thakurgaon	11	12	0.919	0.95	0.983	0.918
103	U	24	24	0.986	0.958	0.955	0.716
104	Panchagarh	7	7	0.977	1	0.995	0.853
105	all	721	6292	0.95	0.898	0.949	0.767

Several districts achieved near-perfect detection rates, including Madaripur (0.978 mAP50-95), Thakurgaon (0.918), Bandarban (0.918), and Narail (0.915), indicating the model’s consistent performance across geographically diverse regions. The numerical digit classes (0–9) showed remarkably consistent performance with mAP50 scores exceeding 0.985 across all digits, highlighting the model’s proficiency in character recognition. However, the analysis revealed certain challenges for lower-performing classes, such as ‘Sa’ (0.346 mAP50-95), ‘Joypurhat’ (0.42), and ‘Pa’ (0.60), which can be attributed to limited training instances and potential class imbalance. These areas present opportunities for future improvement through targeted data augmentation and the addition of new training samples. The model’s high precision score of 0.95 ensures minimal false positives, making it suitable for real-world deployment in automated license plate recognition systems for traffic monitoring, toll collection, and law enforcement applications across Bangladesh. The consistent performance across diverse district codes and character types validates the model’s generalization capability and readiness for practical implementation in the Bangladeshi context.

**Fig 26:**
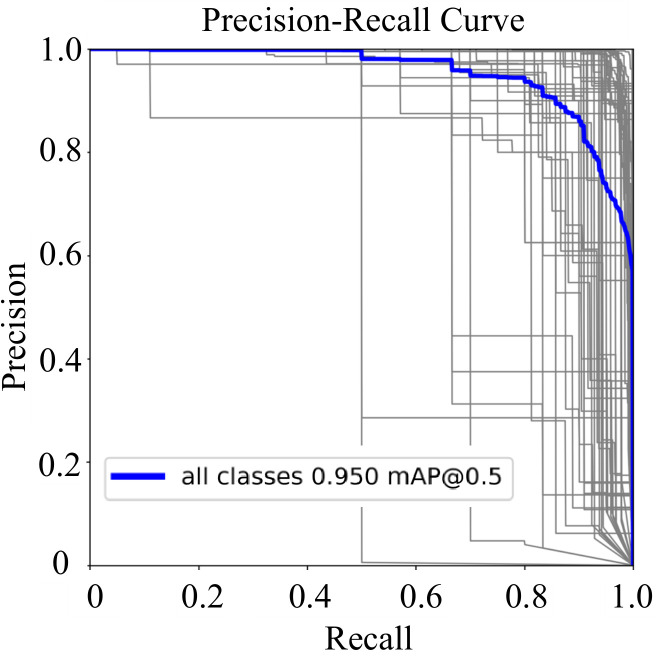
LPNR model test Precision–Recall curve.

**Fig 27:**
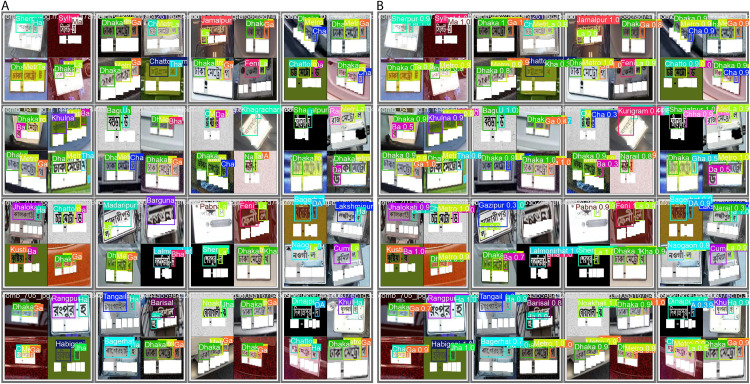
Comparison of LPNR model testing batch: ground truth vs predictions. (a) Testing Batch with Labels. (b) Testing Batch with Predictions.

**Fig 28:**
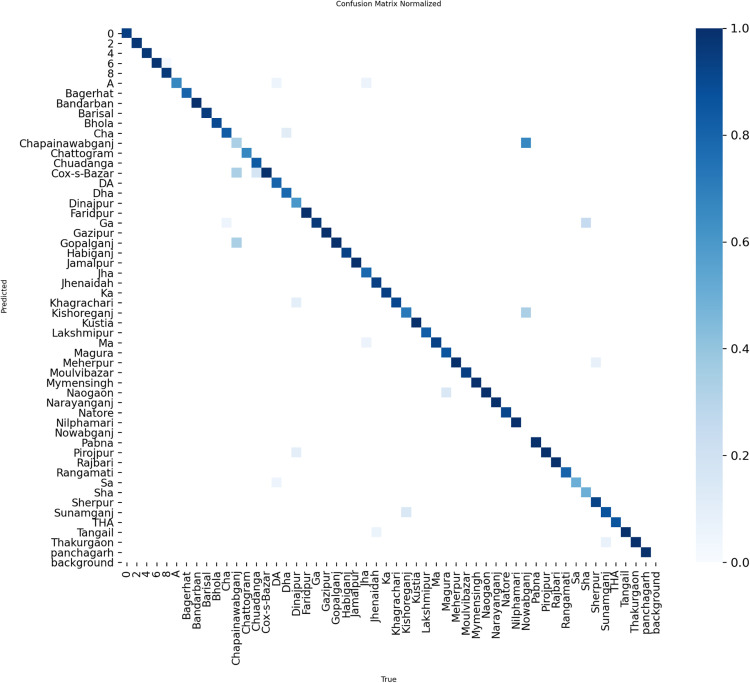
Test set normalized confusion matrix of LPNR model.

Fig 26 presents the Precision–Recall Curve of the testing of the LPNR model, in which the mAP@0.5 is 0.95, indicating a good trade-off between the precision and recall. Fig 27 presents a comparison of the LPNR model testing batch: ground truth vs predictions. Fig 28 presents the normalized confusion matrix of the test set. The confusion matrix demonstrates high classification accuracy, evidenced by a prominent dark blue diagonal line where values approach 1.0 for the majority of classes, including digits, characters, and Bangladeshi district names. Off-diagonal misclassifications are sparse, though minor confusion is observed in complex labels such as “Cox-s-Bazar” and “Khagrachari.”

Fig 27a and 27b show a side-by-side visual validation of the LPNR model’s performance on a sample batch from the test set. The image in Fig 27a represents the **ground truth**, displaying the manually annotated “correct answers” for various license plate elements such as district names (e.g., “Dhaka”, “Sherpur”), headers (“Metro”), and class identifiers. In contrast, Fig 27b illustrates the **model’s predictions**, overlaying detected bounding boxes with their assigned class labels and confidence scores (ranging from 0.3 to 1.0). A comparison between the two figures reveals a high degree of alignment, where the predicted boxes closely match the ground truth in both position and classification. The prevalence of high confidence scores (mostly 0.9 or 1.0) across diverse lighting and plate conditions visually corroborates the statistical success seen in the confusion matrix, confirming the model’s robustness in real-world detection scenarios.

### 3.3 External validation of the proposed cascaded model

The overall cascaded model (VLPDR model) has been externally validated using 150 diverse images collected from both online and offline sources. These images have represented a wide variety of real-world scenarios, including buses, trucks, cars, and motorcycles, each featuring distinct license plates from multiple cities. This external validation has ensured that the proposed model performs reliably across heterogeneous vehicle types, plate styles, and imaging conditions.

Table 10 presents the external validation performances proposed of the VLPDR model on 150 images. The performance evaluation results have demonstrated that the proposed license plate recognition system has achieved highly reliable detection and recognition capabilities. The model has achieved a perfect license plate detection accuracy of 100%, indicating that all license plates in the test dataset have been successfully detected. For license number recognition, the system has achieved 95.33% full-number recognition accuracy, while the character-wise mean accuracy has reached 98.82%, showing strong performance at the individual character level. Furthermore, the Levenshtein-based accuracy of 99.57% has indicated that the predicted license numbers are extremely close to the ground truth values, with only minimal character-level differences.

**Table 10:** Detailed external validation performances on 150 images of the VLPDR model.

Evaluation Metric	Accuracy (%)
License Plate Detection	100.00%
Full Number Recognition (Exact Match)	95.33%
Character-wise Mean Accuracy	98.82%
Levenshtein-based Similarity Accuracy	99.57%

**Fig 29:**
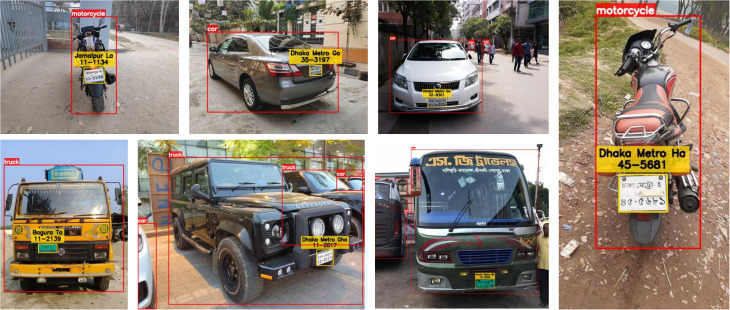
Some correct license plate detection and recognition using the VLPDR model.

**Fig 30:**
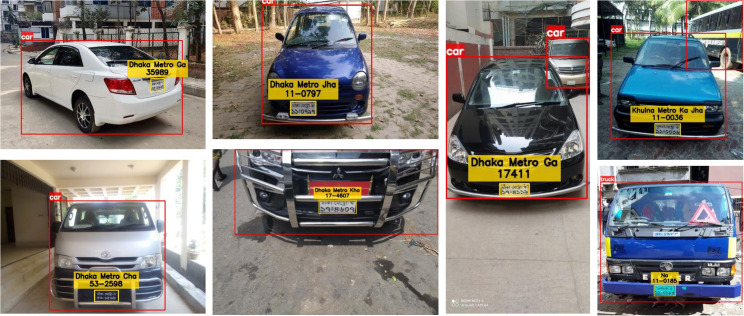
Seven wrong license plate detection and recognition using the VLPDR model.

**Fig 31:**
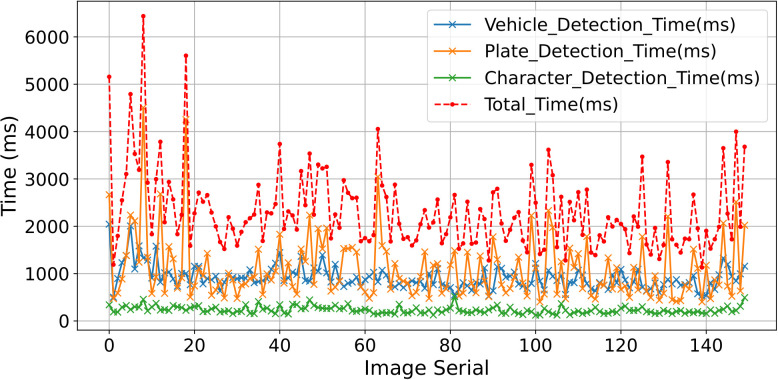
Image-wise execution time in each stage on a laptop without a GPU.

Fig 29 presents some of the perfect detections using the cascaded VLPDR model. Only 7 images have been wrongly detected, but the wrong detections are partial. Fig 30 shows the 7 images that have wrong detection of license numbers. The computational complexity of the proposed model has been analyzed on various devices, including a laptop without a GPU, a computer with a GPU, and the Jetson Orin. Table 11 and Fig 31 illustrate the computational complexity measured on a laptop without a GPU (Processor: Intel^®^ Core™ i5-8265U CPU @ 1.60 GHz (1.80 GHz) with 8.00 GB RAM (7.89 GB usable)).

**Table 11:** Execution time statistics (ms) on a laptop without a GPU.

	Vehicle Detection	Plate Detection	Character Detection	Total Time
Mean	911.39	1091.19	232.92	2311.53
Std	244.09	674.15	74.42	836.39
Min	501.49	403.03	122.61	1130.40
Max	2044.80	4505.91	515.67	6438.13

Table 11 presents the detection time statistics (in milliseconds) of the proposed model on a laptop without a GPU. On average, vehicle detection took 911.39 ms, plate detection took 1091.19 ms, and character detection took 232.92 ms, resulting in a total detection time of 2311.53 ms. The standard deviations indicate variability across images: 244.09 ms for vehicle detection, 674.15 ms for plate detection, 74.42 ms for character detection, and 836.39 ms for total time. Minimum and maximum times ranged from 501.49–2044.80 ms for vehicle detection, 403.03–4505.91 ms for plate detection, 122.61–515.67 ms for character detection, and 1130.40–6438.13 ms for total detection time. Based on the Fig 31, the LPNR model has shown a more stable time consumption performance, and the LPD model has shown more variability during the detection time.

**Fig 32:**
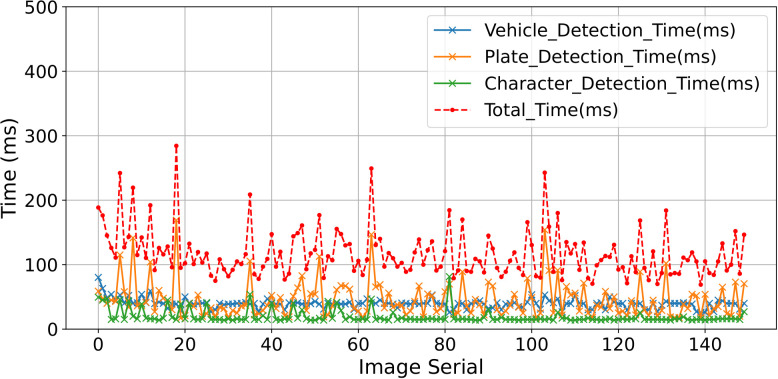
Image-wise execution time in each stage on a GPU-based computer.

Next, Table 12 presents the detection time statistics (in milliseconds) of the proposed model on a GPU-based computer with an AMD Ryzen 9 7900X processor, 16 GB RAM, and an NVIDIA GeForce RTX 3050 GPU. On average, vehicle detection took 39.63 ms, plate detection took 45.18 ms, and character detection took 19.51 ms, resulting in a total detection time of 117.16 ms. The standard deviations indicate variability across images: 7.31 ms for vehicle detection, 28.20 ms for plate detection, 10.44 ms for character detection, and 37.74 ms for total detection time. Minimum and maximum times ranged from 25.98–80.30 ms for vehicle detection, 17.11–168.99 ms for plate detection, 13.99–81.48 ms for character detection, and 69.00–284.31 ms for total detection time. Furthermore, Fig 32 illustrates that the external validation on the GPU-based system has been completed significantly faster compared to the CPU-based system.

**Table 12:** Execution time statistics (ms) on a GPU-based computer.

	Vehicle Detection	Plate Detection	Character Detection	Total Time
Mean	39.63	45.18	19.51	117.16
Std	7.31	28.20	10.44	37.74
Min	25.98	17.11	13.99	69.00
Max	80.30	168.99	81.48	284.31

**Fig 33:**
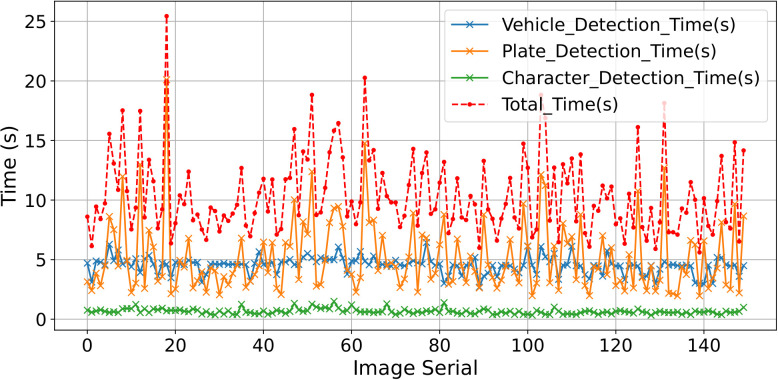
Image-wise execution time in each stage on a Jetson CPU.

The detection time of the VLPDR model on the Jetson CPU has been evaluated for vehicle detection, plate detection, character recognition, and the total processing time, as summarized in Table 13 and Fig 33. The model has achieved a mean vehicle detection time of 4.54 s, a mean plate detection time of 5.15 s, and a mean character detection time of 0.66 s, resulting in an average total inference time of 10.37 s per image. The minimum and maximum times have varied from 2.69 s to 6.44 s for vehicle detection, 1.96 s to 20.10 s for plate detection, 0.34 s to 1.50 s for character detection, and 5.61 s to 25.44 s for the total time, indicating that plate detection has exhibited the highest variability (STD = 3.00 s). Overall, these results demonstrate that the Jetson CPU successfully processed the model, albeit with longer inference times than standard laptop or GPU platforms.

**Table 13:** Execution time statistics (s) on the Jetson CPU.

	Vehicle Detection	Plate Detection	Character Detection	Total Time
Mean	4.535	5.145	0.656	10.367
Std	0.703	3.000	0.219	3.310
Min	2.692	1.961	0.339	5.609
Max	6.439	20.102	1.501	25.439

### 3.4 Comparison of the proposed model

In this subsection, we have compared our proposed model with other YOLO versions and OCR-based models. For the YOLO version comparison, we have considered only Dataset-02, containing images for license plate character detection. For OCR-based comparison, we have used the external validation set containing 150 images.

#### 3.4.1 Comparison with other YOLO versions.

Additionally, we have analyzed the comparative performance of the LPNR model on Dataset-02 using different versions of the YOLO architecture while maintaining the same training, validation, and test data splits. The results are compared against the proposed YOLOv12-based model, as presented in Table 14. The experimental results have demonstrated that YOLOv12 achieves the best overall detection performance, with a validation mAP50 of 0.950 and a test mAP50 of 0.949. Furthermore, YOLOv12 has achieved the highest precision on both the validation set (0.952) and the test set (0.950), while maintaining strong recall of 0.905 and 0.898, respectively. Among the previous YOLO versions, YOLOv8 has exhibited the closest performance with a test mAP50 of 0.936, whereas YOLOv10 has shown the lowest detection capability with a test mAP50 of 0.897. The consistent improvements achieved by YOLOv12 in terms of precision, recall, and mAP50 indicate its superior object localization and recognition capability for Bangladeshi license plate detection and recognition, justifying its selection as the backbone model for the proposed LPNR system.

**Table 14:** Evaluation of multiple versions of the YOLO algorithm on the Dataset-02.

Model Version	Validation Set Result (1438 Images)			Test Set Result (721 Images)		
	Box-P	Box-R	mAP50	Box-P	Box-R	mAP50
YOLOv8	0.946	0.896	0.942	0.937	0.881	0.936
YOLOv9	0.938	0.879	0.934	0.943	0.879	0.938
YOLOv10	0.849	0.833	0.897	0.866	0.811	0.897
YOLOv11	0.926	0.886	0.932	0.933	0.876	0.931
Used Version (YOLOv12)	0.952	0.905	0.950	0.950	0.898	0.949

#### 3.4.2 Comparison with other OCR-based approach.

**Fig 34:**
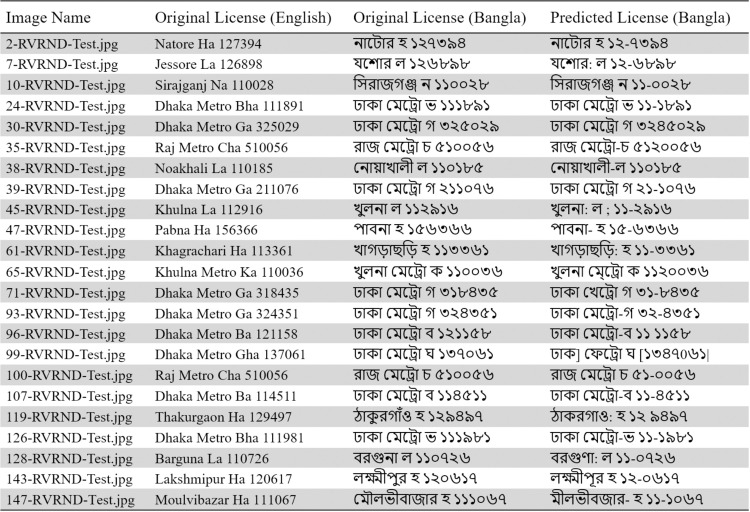
OCR-based correct recognitions (ignoring minor mismatches).

Among the 150 images, the OCR approach has extracted only 23 correct(ignoring minor mismatches) license plate numbers. Fig. 34 presents the correct extraction from the OCR-based approach model. So, the OCR-based approach has only achieved 15.33% accuracy. However, our proposed cascaded model has achieved 95.33% accuracy (Exact Match) as shown in Table 15.

**Table 15:** Performance comparison between the proposed cascaded model and the OCR-based model.

Model	Images	Correct	Incorrect	Accuracy (%)
Proposed Cascaded Model	150	143	7	95.33
OCR-based Model	150	23	127	15.33

## 4. Discussion

In this work, we have developed an accurate and efficient hybrid model by cascading three YOLO-based models for Bangladeshi vehicle license plate detection and license number recognition. In this architecture, one pretrained model and two custom-developed YOLOv12-based models have been used. The pretrained model has been employed to detect vehicles in the input frames. Subsequently, the developed License Plate Detection (LPD) model has been applied to localize the license plate region from the detected vehicle, and the License Plate Number Recognition (LPNR) model has been used to recognize the characters from the localized license plate. This cascading framework has enabled precise vehicle detection, reliable plate localization, and accurate character recognition.

Table 16 presents the training, validation, and testing performance of the two custom YOLO-based models developed for license plate detection (LPD) and license plate number recognition (LPNR). The results demonstrate a substantial improvement in model performance from the first epoch to the final epoch, indicating effective learning and convergence of both models during the training process. For the LPD model, the initial training epoch showed very low performance, with a precision of 0.00117, a recall of 0.18319, and an mAP@50 of 0.00071, reflecting the model’s untrained state at the start of training. However, after completing the training process, the model’s performance has improved significantly, achieving a precision of 0.99259, a recall of 0.96983, and mAP@50 of 0.98057 in the last epoch. This dramatic increase indicates that the model has effectively learned the spatial and contextual features required for accurate license plate localization. The validation results have remained consistent with the final training performance, achieving precision, recall, and mAP@50 values of 0.993, 0.970, and 0.974, respectively. Similarly, the testing results have demonstrated strong generalization capability, with a precision of 0.986, recall of 0.965, and mAP@50 of 0.963, indicating that the model performs reliably on unseen data.

**Table 16:** Training and evaluation performance of the custom YOLO models.

Model	Training (First Epoch)			Training (Last Epoch)			Validation			Testing		
	P	R	mAP@50	P	R	mAP@50	P	R	mAP@50	P	R	mAP@50
LPD	0.00117	0.18319	0.00071	0.99259	0.96983	0.98057	0.993	0.970	0.974	0.986	0.965	0.963
LPNR	0.73846	0.04531	0.03965	0.95110	0.90581	0.94863	0.952	0.905	0.950	0.950	0.898	0.949

A similar training progression has been observed for the LPNR model, although the initial behavior differs slightly due to the complexity of character-level recognition. In the first epoch, the model has achieved a relatively higher precision of 0.73846 but an extremely low recall of 0.04531 and mAP@50 of 0.03965, suggesting that while some predictions were correct, the model initially failed to detect most characters. As training progressed, the model significantly improved, reaching a final training precision of 0.95110, a recall of 0.90581, and an mAP@50 of 0.94863. The validation results have remained highly consistent with the training results, achieving precision, recall, and mAP@50 values of 0.952, 0.905, and 0.950, respectively. Furthermore, the testing performance has also confirmed the robustness of the model, with a precision of 0.950, a recall of 0.898, and mAP@50 of 0.949. Overall, the results presented in Table 16 indicate that both YOLO-based models have achieved strong convergence and stable performance across training, validation, and testing datasets. The minimal performance gap between validation and testing metrics suggests good generalization capability and limited overfitting. Moreover, the LPD model has achieved slightly higher detection accuracy compared to the LPNR model, which is expected because character-level recognition is generally more challenging than object-level detection. These findings confirm the effectiveness of the proposed model in a detection and recognition framework for vehicle license plate.

Table 17 presents the external validation performance of the proposed cascaded license plate detection and recognition framework evaluated on an independent dataset consisting of *n* = 150 samples. The results demonstrate excellent detection and recognition capability of the proposed system. The license plate detection module has achieved a perfect detection accuracy of 100.00%, indicating that all license plates in the external dataset have been successfully localized. For character recognition, the system has obtained a character-wise mean accuracy of 98.82%, which reflects the high reliability of the character detection and classification stage. In addition, the Levenshtein-based similarity accuracy has reached 99.57%, showing that the predicted license plate numbers are extremely close to the ground truth even when minor character mismatches occur. Furthermore, the full number recognition accuracy, which requires an exact match of the entire license plate sequence, has achieved 95.33%. This slightly lower value compared to the character-wise accuracy is expected, since a single character error leads to an incorrect full plate prediction. Nevertheless, the overall results confirm the strong robustness and effectiveness of the proposed cascaded architecture in real-world license plate recognition scenarios.

**Table 17:** External validation performance and inference time of the proposed cascaded model.

Category	Evaluation Metric / Platform	Value
External Validation Results (*n* = 150)		
Performance	License Plate Detection Accuracy	100.00%
Performance	Character-wise Mean Accuracy	98.82%
Performance	Levenshtein-based Similarity Accuracy	99.57%
Performance	Full Number Recognition (Exact Match)	95.33%
Inference Time Comparison		
Latency	Laptop (CPU only)	2311.53 ms
Latency	Desktop PC (GPU-enabled)	284.31 ms
Latency	Jetson CPU (without GPU)	25.439 s

The Table 17 also reports the inference latency of the proposed system across different hardware platforms to evaluate its computational efficiency. On a standard laptop running only on CPU, the system requires 2311.53 ms per inference, which demonstrates moderate processing speed without dedicated acceleration. When executed on a GPU-enabled desktop PC, the inference time has been significantly reduced to 284.31 ms, highlighting the advantage of GPU parallelization for deep learning inference tasks. In contrast, the Jetson platform operating without GPU acceleration has shown a substantially higher latency of 25.439 s, indicating that CPU-only embedded environments may face performance limitations for real-time deployment. Overall, the results suggest that the proposed cascaded model can achieve near real-time performance on GPU-supported systems while maintaining high recognition accuracy across different evaluation metrics.

Table 1 summarizes several recent studies on Bangladeshi license plate detection and recognition. Most existing works have achieved promising detection performance using different YOLO variants, such as YOLOv4 [18,19], YOLOv7 [5,20], YOLOv8 [23,24], and YOLOv10 [25]. However, the recognition stage in many of these studies has relied either on OCR-based systems or on models trained with a relatively small number of character classes. For example, CNN + SVM with only 14 classes was used in [14], while HOG + SVM with 18 classes was applied in [15]. Similarly, YOLO-based recognition with 27 and 30 classes was reported in [18] and [19], respectively. Even more recent approaches, such as [21] and [5], have considered around 60 classes. In contrast, the proposed model incorporates 104 recognition classes to cover a much broader range of Bangladeshi license plate components, including city codes, vehicle type identifiers, and numeric digits. Despite this significantly larger class space, the proposed LPNR model has achieved strong performance with a testing precision of 0.95, recall of 0.898, and mAP@50 of 0.949, demonstrating that the model can effectively handle a more complex recognition problem while maintaining competitive accuracy.

In terms of overall system performance, the proposed cascaded framework has also shown competitive results compared with previous studies. For instance, recognition accuracies of 96.31%, 97%, and 95% have been reported in [5,18], and [21], respectively, while some OCR-based systems have achieved significantly lower accuracy, such as 78% in [16] and 22%−−73% in [17]. The proposed system has achieved 100% license plate detection accuracy and 95.33% full number recognition accuracy during external validation on 150 images. Additionally, the character-wise mean accuracy has reached 98.82%, and the Levenshtein-based similarity accuracy has achieved 99.57%, indicating that most predicted license numbers closely match the ground truth sequences. Given that the proposed model supports more than 100 recognition classes and avoids traditional OCR engines, these results demonstrate that the cascaded YOLO-based approach offers a more comprehensive and practical solution for Bangladeshi license plate recognition while maintaining competitive accuracy compared with existing methods.

## 5. Conclusion

This study has presented a cascaded deep learning framework for vehicle license plate detection and recognition tailored to the structure of Bangladeshi license plates. The proposed VLPDR system integrates three YOLOv12 models in a sequential architecture to perform vehicle detection, license plate localization, and character-level recognition. Unlike many existing approaches that rely on traditional OCR engines or a limited number of recognition classes, the proposed model incorporates 104 character classes to represent a wider range of Bangladeshi license plate components. This design improves the capability of the system to handle the diverse textual structure of license plates while avoiding common OCR-related limitations associated with complex Bangla scripts. Experimental results demonstrate that the proposed models achieve strong detection and recognition performance. The license plate detection model has achieved testing precision, recall, and mAP@50 values of 0.986, 0.965, and 0.963, respectively, while the license plate number recognition model has achieved testing precision, recall, and mAP@50 values of 0.95, 0.898, and 0.949. Furthermore, external validation on 150 images has shown 100% license plate detection accuracy and 95.33% full number recognition accuracy, with a character-wise mean accuracy of 98.82% and a Levenshtein-based similarity accuracy of 99.57%. The model has also been evaluated on different hardware platforms, demonstrating its feasibility for practical deployment. Overall, the results indicate that the proposed cascaded YOLO-based framework provides a robust and scalable solution for automated vehicle license plate detection and recognition in Bangladesh and similar environments.

Future work will focus on validating the proposed framework on larger and more diverse external datasets under challenging conditions, such as low illumination, adverse weather, motion blur, and occlusions. In addition, strategies to better address class imbalance and comparisons with lightweight real-time models will be investigated to further improve deployment efficiency and robustness.
