# Supplementary material for: Cascaded YOLO-based approach for Bangladeshi license plate identification and number recognition
Source: PLoS One. 2026 Aug 10;21(8):e0355858. doi: 10.1371/journal.pone.0355858 (PMC13456284; doi:10.1371/journal.pone.0355858)
Supplement: Supplementary file 1 [file pone.0355858.s001.docx]

**Editor**

**Comments and Response**

**Comment 1:** *The novelty and contribution of the study should be clarified more explicitly. A cascaded YOLO-based framework is not, by itself, sufficiently novel. The manuscript should clearly explain what is technically new in the proposed 104-class Bangladeshi license plate recognition approach, especially in comparison with existing YOLO-based and OCR-based license plate recognition systems.*

**Response:**

We sincerely thank the reviewer for this valuable comment. We agree that a cascaded YOLO-based framework alone does not constitute sufficient novelty. Therefore, we have revised the manuscript to clearly emphasize the following contributions:

1. **104-Class Bangladeshi License Plate Recognition:** The proposed framework supports 104 license plate classes, which is substantially higher than existing studies. Based on our literature review, the closest related work considered only 60 classes, limiting its applicability to the diverse categories of Bangladeshi license plates.
2. **OCR-Free Recognition:** Unlike conventional ALPR systems, the proposed method directly recognizes license plate classes without using OCR. This is particularly beneficial for Bangla license plates, where OCR systems often struggle with complex conjunct consonants (*Jukto Borno*), leading to recognition errors.
3. **Cross-Platform Deployment Evaluation:** The framework is evaluated on CPU-based systems, GPU-based systems, and an NVIDIA Jetson edge device, demonstrating its effectiveness and practical deployability across different computing environments.

To better highlight these aspects, we have revised the Introduction and Contribution sections of the manuscript. To the best of our knowledge, no previous study has reported a Bangladeshi license plate recognition framework that simultaneously incorporates a 104-class recognition scheme, OCR-free recognition, and validation across both desktop and edge-computing platforms. The revised contributions can be found on lines 111-134 within the manuscript.

**Comment 2:** *The choice of YOLOv12 should be technically justified. The manuscript should include comparative experiments with relevant YOLO versions, such as YOLOv8, YOLOv9, YOLOv10, or YOLOv11, using the same dataset split, training protocol, evaluation metrics, and hardware conditions.*

**Response:**

We are very grateful to the editor for this insightful suggestion, which could enhance the manuscript's quality. We have mainly chosen YOLOv12 due to its theoretical impact. After your suggestion, we have evaluated the performance of other versions of the YOLO algorithm using the same dataset split and setup. We have revised our manuscript with a new subsection:

3.2.1 Justification of Choosing YOLOv12:

In this work, we have used the base architecture of YOLOv12 as it has successfully 246 integrated an attention-centric design into the YOLO framework, achieving a state- 247 of-the-art latency-accuracy trade-off [27]. Empirical evaluations in the foundational 248 study demonstrate its superiority; for example, YOLOv12-N achieves 40.6% mAP with 249 an inference latency of just 1.64 ms, outperforming YOLOv10-N and YOLOv11-N 250 by 2.1% and 1.2% mAP, respectively [1]. Furthermore, the architecture is highly 251 resource-efficient—variants like YOLOv12-S operate 42% faster than advanced end-to- 252 end detectors like RT-DETRv2-R18, utilizing only 36% of the computation and 45% of 253 the parameters [27]. By utilizing a novel area attention module and Residual Efficient 254 Layer Aggregation Networks (R-ELAN), YOLOv12 provides an optimal foundation for 255 our methodology, allowing us to harness the robust modeling capabilities of attention 256 mechanisms while exceeding the real-time inference efficiency of traditional CNN-based 257 detectors.

As you suggested, we have performed multiple versions of the YOLO algorithms on the License Plate Number Recognition (LPNR) dataset. So, we have revised our manuscript with this information in section 4.4.1: Comparison with other YOLO Versions.

Additionally, we have analyzed the comparative performance of the LPNR model on Dataset-02 using different versions of the YOLO architecture while maintaining the same training, validation, and test data splits. The results are compared against the proposed YOLOv12-based model, as presented in Table 1. The experimental results have demonstrated that YOLOv12 has achieved the best overall detection performance, obtaining a validation mAP50 of 0.950 and a test mAP50 of 0.949. Furthermore, YOLOv12 has attained the highest precision values on both the validation set (0.952) and the test set (0.950), while maintaining a strong recall of 0.905 and 0.898, respectively. Among the previous YOLO versions, YOLOv8 has exhibited the closest performance with a test mAP50 of 0.936, whereas YOLOv10 has shown the lowest detection capability with a test mAP50 of 0.897. The consistent improvements achieved by YOLOv12 in terms of precision, recall, and mAP50 indicate its superior object localization and recognition capability for Bangladeshi license plate detection and recognition, justifying its selection as the backbone model for the proposed LPNR system.

Table 1: Evaluation of Multiple versions of the YOLO algorithm on the LPNR dataset

| Model Version | Validation Set Result (1438 Images) | | | Test Set Result (721 Images) | | |
| --- | --- | --- | --- | --- | --- | --- |
|  | Box-P | Box-R | mAP50 | Box-P | Box-R | mAP50 |
| YOLOv8 | 0.946 | 0.896 | 0.942 | 0.937 | 0.881 | 0.936 |
| YOLOv9 | 0.938 | 0.879 | 0.934 | 0.943 | 0.879 | 0.938 |
| YOLOv10 | 0.849 | 0.833 | 0.897 | 0.866 | 0.811 | 0.897 |
| YOLOv11 | 0.926 | 0.886 | 0.932 | 0.933 | 0.876 | 0.931 |
| YOLOv12 | 0.952 | 0.905 | 0.950 | 0.950 | 0.898 | 0.949 |

**Comment 3:** *The experimental comparison should be strengthened. Comparisons should not rely only on literature-level summaries. The authors should compare the proposed model with OCR-based and YOLO-based baseline methods on the same test set wherever possible.*

**Response:**

We really appreciate the editor's valuable comment. As suggested, we tested an OCR-based approach that combines License Plate Detection (LPD) for detecting license plates and easy OCR for recognizing Bangla license numbers. Then we have compared the results with our proposed cascaded model’s results. For this evaluation, we have considered 150 images used in external validation to enable better comparison.

Among the 150 images, the OCR approach only succeeded in extracting 23 almost correct license plate numbers. Table 2 presents the correct extraction from the OCR-based approach model. So, the OCR-based approach has only achieved 15.33% accuracy. However, our proposed cascaded model has achieved 95.33% accuracy (Exact Match). So, we have revised our manuscript and enriched it with this comparison result. The full result on 150 External Validated Images of the proposed cascaded model and OCR-based approach can be found in this link:

<https://github.com/prosensarker4/BLPD/tree/main/External%20Validated%20Images>

Table 2: OCR-based Almost Correct Predictions.

| Image Name | Original License (English) | Original License (Bangla) | Predicted License (Bangla) |
| --- | --- | --- | --- |
| 2-RVRND-Test.jpg | Natore Ha 127394 | নাটোর হ ১২৭৩৯৪ | নাটোর হ ১২-৭৩৯৪ |
| 7-RVRND-Test.jpg | Jessore La 126898 | যশোর ল ১২৬৮৯৮ | যশোর: ল ১২-৬৮৯৮ |
| 10-RVRND-Test.jpg | Sirajganj Na 110028 | সিরাজগঞ্জ ন ১১০০২৮ | সিরাজগঞ্জ ন ১১-০০২৮ |
| 24-RVRND-Test.jpg | Dhaka Metro Bha 111891 | ঢাকা মেট্রো ভ ১১১৮৯১ | ঢাকা মেট্রো ভ ১১-১৮৯১ |
| 30-RVRND-Test.jpg | Dhaka Metro Ga 325029 | ঢাকা মেট্রো গ ৩২৫০২৯ | ঢাকা মেট্রো গ ৩২৪৫০২৯ |
| 35-RVRND-Test.jpg | Raj Metro Cha 510056 | রাজ মেট্রো চ ৫১০০৫৬ | রাজ মেট্রো-চ ৫১২০০৫৬ |
| 38-RVRND-Test.jpg | Noakhali La 110185 | নোয়াখালী ল ১১০১৮৫ | নোয়াখালী-ল ১১০১৮৫ |
| 39-RVRND-Test.jpg | Dhaka Metro Ga 211076 | ঢাকা মেট্রো গ ২১১০৭৬ | ঢাকা মেট্রো গ ২১-১০৭৬ |
| 45-RVRND-Test.jpg | Khulna La 112916 | খুলনা ল ১১২৯১৬ | খুলনা: ল ; ১১-২৯১৬ |
| 47-RVRND-Test.jpg | Pabna Ha 156366 | পাবনা হ ১৫৬৩৬৬ | পাবনা- হ ১৫-৬৩৬৬ |
| 61-RVRND-Test.jpg | Khagrachari Ha 113361 | খাগড়াছড়ি হ ১১৩৩৬১ | খাগড়াছড়ি: হ ১১-৩৩৬১ |
| 65-RVRND-Test.jpg | Khulna Metro Ka 110036 | খুলনা মেট্রো ক ১১০০৩৬ | খুলনা মে্ট্রো ক ১১২০০৩৬ |
| 71-RVRND-Test.jpg | Dhaka Metro Ga 318435 | ঢাকা মেট্রো গ ৩১৮৪৩৫ | ঢাকা খেট্রো গ ৩১-৮৪৩৫ |
| 93-RVRND-Test.jpg | Dhaka Metro Ga 324351 | ঢাকা মেট্রো গ ৩২৪৩৫১ | ঢাকা মেট্রো-গ ৩২-৪৩৫১ |
| 96-RVRND-Test.jpg | Dhaka Metro Ba 121158 | ঢাকা মেট্রো ব ১২১১৫৮ | ঢাকা মেট্রো-ব ১১ ১১৫৮ |
| 99-RVRND-Test.jpg | Dhaka Metro Gha 137061 | ঢাকা মেট্রো ঘ ১৩৭০৬১ | ঢাক] ফেট্রো ঘ [১৩৪৭0৬১\| |
| 100-RVRND-Test.jpg | Raj Metro Cha 510056 | রাজ মেট্রো চ ৫১০০৫৬ | রাজ মেট্রো চ ৫১-০০৫৬ |
| 107-RVRND-Test.jpg | Dhaka Metro Ba 114511 | ঢাকা মেট্রো ব ১১৪৫১১ | ঢাকা মেট্রো-ব ১১-৪৫১১ |
| 119-RVRND-Test.jpg | Thakurgaon Ha 129497 | ঠাকুরগাঁও হ ১২৯৪৯৭ | ঠাকরগাও: হ ১২ ৯৪৯৭ |
| 126-RVRND-Test.jpg | Dhaka Metro Bha 111981 | ঢাকা মেট্রো ভ ১১১৯৮১ | ঢাকা মেট্রো-ভ ১১-১৯৮১ |
| 128-RVRND-Test.jpg | Barguna La 110726 | বরগুনা ল ১১০৭২৬ | বরগুণা: ল ১১-০৭২৬ |
| 143-RVRND-Test.jpg | Lakshmipur Ha 120617 | লক্ষ্মীপুর হ ১২০৬১৭ | লক্ষ্মীপূর হ ১২-০৬১৭ |
| 147-RVRND-Test.jpg | Moulvibazar Ha 111067 | মৌলভীবাজার হ ১১১০৬৭ | মীলভীবজার- হ ১১-১০৬৭ |

So, Table 3 presents the comparison between the proposed cascaded model and the ORC-based approach results:

Table 3: Comparison between the Proposed Cascaded Model and ORC-based Approach Results

| Model | Images | Correct | Incorrect | Accuracy |
| --- | --- | --- | --- | --- |
| Proposed Cascaded Model | 150 | 143 | 7 | 95.33% |
| OCR-based Model | 150 | 23 | 127 | 15.33% |

**Comment 4:** *The dataset description requires substantial improvement. The manuscript should provide clearer information on image sources, camera conditions, lighting conditions, day/night images, weather variation, vehicle types, geographical diversity, city coverage, dataset splits, class distribution, and external validation images.*

**Response:**

We are really thankful to the editor for this comment. In our dataset description, we have already described the dataset sources and cities. However, according to your suggestion, we have revised our dataset description section (3.1 Dataset Description).

Dataset-01: <https://github.com/prosensarker4/BLPD/tree/main/LPD_Dataset>

Dataset-02: <https://github.com/prosensarker4/BLPD/tree/main/LPNR_Dataset>

External Validation Images:

<https://github.com/prosensarker4/BLPD/tree/main/External%20Validated%20Images/Input>

**Comment 5:** *The class imbalance in Dataset 02 should be discussed in detail. Per-class precision, recall, F1-score, and confusion matrices should be provided, particularly for visually similar Bangla characters and classes that may be difficult to distinguish.*

**Response:**

We are really thankful to the editor for this comment. We absolutely agree that Dataset 02 has a noticeable class imbalance. The table given below shows the class names and their counts. We have revised our dataset description section to include information on the class imbalances in Dataset-02. For your concern, we have already provided the per-class Confusion matrix, Precision, Recall, mAP50, and mAP50-95. See Table 8, Fig. 25, and Fig. 28 in the revised manuscript.

Table 4: Class Names and their quantity

| **Name** | **Count** | **Name** | **Count** | **Name** | **Count** | **Name** | **Count** | **Name** | **Count** |
| --- | --- | --- | --- | --- | --- | --- | --- | --- | --- |
| 0 | 2778 | Chandpur | 46 | Gopalganj | 114 | Madaripur | 100 | Patuakhali | 152 |
| 1 | 7396 | Chapainawabganj | 79 | Ha | 2179 | Magura | 65 | Pirojpur | 120 |
| 2 | 3297 | Chatto | 603 | Habiganj | 120 | Manikganj | 125 | Raj | 221 |
| 3 | 3110 | Chattogram | 97 | Ja | 236 | Meherpur | 51 | Rajbari | 101 |
| 4 | 2655 | Chha | 178 | Jamalpur | 150 | Metro | 6292 | Rajshahi | 88 |
| 5 | 2610 | Chuadanga | 84 | Jessore | 289 | Moulvibazar | 191 | Rangamati | 73 |
| 6 | 2223 | Cox-s Bazar | 47 | Jha | 211 | Munshiganj | 2 | Rangpur | 144 |
| 7 | 2431 | Cox-s-Bazar | 58 | Jhalokati | 96 | Mymensingh | 203 | Sa | 53 |
| 8 | 2411 | Cumilla | 81 | Jhenaidah | 162 | Na | 323 | Satkhira | 115 |
| 9 | 2071 | Da | 402 | Joypurhat | 45 | Naogaon | 123 | Sha | 131 |
| A | 184 | DA | 141 | Ka | 188 | Narail | 60 | Shariatpur | 86 |
| Ba | 409 | Dha | 136 | Kha | 264 | Narayanganj | 83 | Sherpur | 85 |
| Bagerhat | 37 | Dhaka | 4947 | Khagrachari | 151 | Narsingdi | 101 | Sirajganj | 139 |
| Bagura | 126 | Dinajpur | 82 | Khulna | 566 | Natore | 184 | Sunamganj | 120 |
| Bandarban | 62 | E | 133 | Kishoreganj | 117 | Netrokona | 65 | Sylhet | 250 |
| Barguna | 70 | Faridpur | 57 | Kurigram | 113 | Nilphamari | 84 | Ta | 713 |
| Barisal | 217 | Feni | 141 | Kustia | 45 | Noakhali | 140 | Tangail | 292 |
| Bha | 306 | Ga | 2853 | La | 2137 | Nowabganj | 11 | Tha | 405 |
| Bhola | 105 | Gaibandha | 54 | Lakshmipur | 128 | Pa | 76 | THA | 179 |
| Brahmanbaria | 68 | Gazipur | 106 | Lalmonirhat | 102 | Pabna | 144 | Thakurgaon | 123 |
| Cha | 430 | Gha | 652 | Ma | 201 | panchagarh | 73 | U | 188 |

**Comment 6:** *The recognition pipeline should be explained more rigorously. The manuscript should clarify whether simple left-to-right sorting is sufficient for Bangladeshi plate structures, including two-line plates and the ordering of city, metro/type, and number components.*

**Response:**

We cordially thank the editor for this insightful comment. We have already explained the pipeline in detail in our manuscript, in section 3.4, "Proposed VLPDR Model Architecture." In this section, we have explained that three YOLO-based models have been cascaded, where 1^st^ model detects the vehicle, 2^nd^ model localizes the license plate, and the 3^rd^ mode recognize the license plate number.

The second concern of this comment is the simple left-to-right sorting of the Bangladeshi license plate. We completely agree that Bangladeshi vehicle license plates contain a two-line structure in which the first line has the city, metropolitan, and vehicle type information, and the second line has the vehicle class number (2 digits) and a unique number (4 digits). So, we have revised our code arrangement to match the two-line structure of the Bangladeshi license plate. We have also revised the associated figures (Figs. 29 and 30), which show the changes. For your concern we have attached an example figure here too.

Fig. 01: Proposed Model Output with Two-line plate structure.

**Comment 7:** *The real-time performance claim should be revised or supported with stronger evidence. The authors should report FPS, latency, model size, computational cost, and hardware details. It should be clearly stated whether inference was performed using CPU, GPU, Jetson GPU, TensorRT, or other acceleration settings. If some reported results are not real-time, the claim should be moderated accordingly.*

**Response:**

We have completely agreed with this comment that more evidence is needed to claim real-time performance. So, we have removed the phrase from our manuscript.

**Comment 8:** *Table 1 should be revised. It should include, for each reviewed study, the method used, dataset name, dataset size, language or plate type, main advantages, main disadvantages, and key performance metrics. The comparison should also discuss relevant works in other languages or plate systems to better contextualize the efficiency of the proposed model.*

**Response:**
We thank the Editor for this valuable suggestion. Table 1 is intended to provide a focused review of studies on Bangladeshi license plate detection and recognition; therefore, the comparison is limited to works involving Bangla license plates used in Bangladesh. Unlike many international benchmarks, most of the reviewed studies employed self-collected datasets and did not utilize publicly available benchmark datasets. Consequently, dataset names were either not reported or were unavailable for several studies, making a comprehensive comparison of dataset characteristics challenging.

To address the Editor’s concern and improve the clarity of the literature review, we have revised Table 1 by adding a new **“Disadvantage”** column that highlights the key shortcomings of each study.

Regarding studies from other languages or license plate systems, the scope of this work is limited to Bangladeshi license plates. Since license plate layouts, dimensions, fonts, and character sets vary significantly across countries, models developed for other license plate systems are not directly comparable to those designed for Bangladeshi plates. Therefore, we have restricted the literature review to studies conducted on Bangladeshi license plates to ensure a fair and relevant comparison.

**Comment 9:** *The manuscript should include more recent references where relevant, including studies published in 2026 if directly related to license plate recognition, YOLO-based recognition, OCR-free recognition, or real-time intelligent transportation/urban surveillance applications.*

**Response:**
We appreciate the editor’s comment. We have revised our manuscript with newly published works (references 26 and 27 in the manuscript) in 2026 related to this work. For your consideration, we have attached the snippet of the new citations added to Table 1 in our revised manuscript.

**Comment 10:** *The general YOLO background section should be shortened. Instead of providing an extended generic explanation of YOLO, the manuscript should give more implementation-specific details, including hyperparameters, augmentation strategy, image size, batch size, number of epochs, optimizer, learning rate, confidence threshold, NMS threshold, early stopping criteria, and training/validation settings.*

**Response:**
Thank you for your technical comment. We have revised our manuscript with a table (Table 5) that provides the experimental settings. For your convenience, we have shared the table below as a snip.

**Comment 11:** *The conclusion should be revised to include a concise paragraph summarizing future work. This may include broader external validation, deployment under challenging environmental conditions, improved treatment of class imbalance, and comparison with lightweight real-time models.*

**Response:**
Thank you for your valuable comment. We have revised our conclusion according to your suggestions.

**Comment 12:** *The English language and journal style should be revised carefully. Phrases such as “In this thesis” and repeated or awkward constructions such as excessive “has been” usage should be corrected. The manuscript should be presented consistently as a journal article.*

**Response:**
Thank you for your comment. We have revised the phrase “*In this thesis*” and the use of excessive “*has been*” in your revised manuscript.

**Reviewer-1**

**Comments and Response**

**Comment 1:** *Table 1 did not mention the advantages and disadvantages of each method used in each study, in addition to the name and size of the dataset used.*

**Response:**
We thank the reviewer for this valuable comment. Table 1 already includes the methods or algorithms used in each study (Column 3). However, the names of the datasets were not consistently reported in several of the reviewed studies; therefore, they could not be included for all entries. To address the reviewer's concern and improve the clarity and completeness of the literature review, we have revised Table 1 by adding a new "**Disadvantage**" column that summarizes the main limitations of each study.

**Comment 2:** *Why wasn't the submitted work compared with similar works in other languages to ensure the efficiency of the proposed model?*

**Response:**
We appreciate the reviewer for his/her comment. The scope of this work is specifically focused on Bangladeshi license plates. Direct comparison with studies developed for other languages/countries is not appropriate because license plate layouts, dimensions, fonts, scripts, and character sets differ significantly across regions. Furthermore, character-level annotation schemes are language-dependent, and the number, type, and representation of character classes vary from one license plate system to another. As a result, recognition models are trained and evaluated under different annotation settings, making direct performance comparisons potentially misleading. Therefore, comparisons with models developed for other license plate systems would not provide a fair or meaningful evaluation. For this reason, we have limited the literature review and performance comparison to studies conducted on Bangladeshi license plates, ensuring that the comparisons are relevant and performed under similar conditions.

**Comment 3:** *Future actions can be summarized in a paragraph and included in the conclusions.*

**Response:**
Thank you for your valuable comment. We have revised our conclusion in accordance with your suggestions.

**Comment 4:** *Adding other references published in 2026.*

**Response:**
We appreciate the reviewer’s comment. We have revised our manuscript with newly published works (references 26 and 27 in the manuscript) in 2026 related to this work.

**Reviewer-2**

**Comments and Response**

**Comment 1:** *The novelty should be explained more clear; cascaded YOLO structure is not very new, so authors should emphasize the real contribution of 104-class Bangladeshi plate recognition without OCR.*

**Response:**
We sincerely thank the reviewer for this valuable comment. We agree that a cascaded YOLO-based framework alone does not constitute sufficient novelty. Therefore, we have revised the manuscript to clearly emphasize the following contributions:

1. **104-Class Bangladeshi License Plate Recognition:** The proposed framework supports 104 license plate classes, which is substantially higher than existing studies. Based on our literature review, the closest related work considered only 60 classes, limiting its applicability to the diverse categories of Bangladeshi license plates.
2. **OCR-Free Recognition:** Unlike conventional ALPR systems, the proposed method directly recognizes license plate classes without using OCR. This is particularly beneficial for Bangla license plates, where OCR systems often struggle with complex conjunct consonants (*Jukto Borno*), leading to recognition errors.
3. **Cross-Platform Deployment Evaluation:** The framework is evaluated on CPU-based systems, GPU-based systems, and an NVIDIA Jetson edge device, demonstrating its effectiveness and practical deployability across different computing environments.

To better highlight these aspects, we have revised the Introduction and Contribution sections of the manuscript. To the best of our knowledge, no previous study has reported a Bangladeshi license plate recognition framework that simultaneously incorporates a 104-class recognition scheme, OCR-free recognition, and validation across both desktop and edge-computing platforms. The revised contributions can be found on lines 111-134 within the manuscript.

**Comment 2:** *The reason of selecting YOLOv12 should be justified, and comparison with YOLOv8, YOLOv9, YOLOv10 or YOLOv11 should be added under same dataset and training settings.*
**Response:**

We are very grateful to the reviewer for this insightful suggestion, which could enhance the manuscript's quality. We have mainly chosen YOLOv12 due to its theoretical impact. After your suggestion, we have evaluated the performance of other versions of the YOLO algorithm using the same dataset split and setup. We have revised our manuscript with a new subsection:

3.2.1 Justification of Choosing YOLOv12:

In this work, we have used the base architecture of YOLOv12 as it has successfully 246 integrated an attention-centric design into the YOLO framework, achieving a state- 247 of-the-art latency-accuracy trade-off [27]. Empirical evaluations in the foundational 248 study demonstrate its superiority; for example, YOLOv12-N achieves 40.6% mAP with 249 an inference latency of just 1.64 ms, outperforming YOLOv10-N and YOLOv11-N 250 by 2.1% and 1.2% mAP, respectively [1]. Furthermore, the architecture is highly 251 resource-efficient—variants like YOLOv12-S operate 42% faster than advanced end-to- 252 end detectors like RT-DETRv2-R18, utilizing only 36% of the computation and 45% of 253 the parameters [27]. By utilizing a novel area attention module and Residual Efficient 254 Layer Aggregation Networks (R-ELAN), YOLOv12 provides an optimal foundation for 255 our methodology, allowing us to harness the robust modeling capabilities of attention 256 mechanisms while exceeding the real-time inference efficiency of traditional CNN-based 257 detectors.

As you suggested, we have performed multiple versions of the YOLO algorithms on the License Plate Number Recognition (LPNR) dataset. So, we have revised our manuscript with this information in section 4.4.1: Comparison with other YOLO Versions.

Additionally, we have analyzed the comparative performance of the LPNR model on Dataset-02 using different versions of the YOLO architecture while maintaining the same training, validation, and test data splits. The results are compared against the proposed YOLOv12-based model, as presented in Table 1. The experimental results have demonstrated that YOLOv12 has achieved the best overall detection performance, obtaining a validation mAP50 of 0.950 and a test mAP50 of 0.949. Furthermore, YOLOv12 has attained the highest precision values on both the validation set (0.952) and the test set (0.950), while maintaining a strong recall of 0.905 and 0.898, respectively. Among the previous YOLO versions, YOLOv8 has exhibited the closest performance with a test mAP50 of 0.936, whereas YOLOv10 has shown the lowest detection capability with a test mAP50 of 0.897. The consistent improvements achieved by YOLOv12 in terms of precision, recall, and mAP50 indicate its superior object localization and recognition capability for Bangladeshi license plate detection and recognition, justifying its selection as the backbone model for the proposed LPNR system.

Table 1: Evaluation of Multiple versions of the YOLO algorithm on the LPNR dataset

| Model Version | Validation Set Result (1438 Images) | | | Test Set Result (721 Images) | | |
| --- | --- | --- | --- | --- | --- | --- |
|  | Box-P | Box-R | mAP50 | Box-P | Box-R | mAP50 |
| YOLOv8 | 0.946 | 0.896 | 0.942 | 0.937 | 0.881 | 0.936 |
| YOLOv9 | 0.938 | 0.879 | 0.934 | 0.943 | 0.879 | 0.938 |
| YOLOv10 | 0.849 | 0.833 | 0.897 | 0.866 | 0.811 | 0.897 |
| YOLOv11 | 0.926 | 0.886 | 0.932 | 0.933 | 0.876 | 0.931 |
| YOLOv12 | 0.952 | 0.905 | 0.950 | 0.950 | 0.898 | 0.949 |

**Comment 3:** *Dataset description and validation should be improved; image sources, camera conditions, day/night, weather, vehicle types, split details and more external images from different cities should be given.*

**Response:**

We are really thankful to the reviewer for this comment. In our dataset description, we have already described the dataset sources and cities. However, according to your suggestion, we have revised our dataset description section (3.1 Dataset Description). And the full dataset can be found here:

Dataset-01: <https://github.com/prosensarker4/BLPD/tree/main/LPD_Dataset>

Dataset-02: <https://github.com/prosensarker4/BLPD/tree/main/LPNR_Dataset>

External Validation Images:

<https://github.com/prosensarker4/BLPD/tree/main/External%20Validated%20Images/Input>

**Comment 4:** *Dataset 02 has visible class imbalance, but this is not discussed enough; per-class precision, recall and confusion matrix should be given especially for similar Bangla characters.*

**Response:**

We are really thankful to the reviewer for this comment. We absolutely agree that Dataset 02 has a noticeable class imbalance. The table given below shows the class names and their counts. We have revised our dataset description section to include information on the class imbalances in Dataset-02. For your concern, we have already provided the per-class Confusion matrix, Precision, Recall, mAP50, and mAP50-95. See Table 8, Fig. 25, and Fig. 28 in the revised manuscript.

Table 4: Class Names and their quantity

| **Name** | **Count** | **Name** | **Count** | **Name** | **Count** | **Name** | **Count** | **Name** | **Count** |
| --- | --- | --- | --- | --- | --- | --- | --- | --- | --- |
| 0 | 2778 | Chandpur | 46 | Gopalganj | 114 | Madaripur | 100 | Patuakhali | 152 |
| 1 | 7396 | Chapainawabganj | 79 | Ha | 2179 | Magura | 65 | Pirojpur | 120 |
| 2 | 3297 | Chatto | 603 | Habiganj | 120 | Manikganj | 125 | Raj | 221 |
| 3 | 3110 | Chattogram | 97 | Ja | 236 | Meherpur | 51 | Rajbari | 101 |
| 4 | 2655 | Chha | 178 | Jamalpur | 150 | Metro | 6292 | Rajshahi | 88 |
| 5 | 2610 | Chuadanga | 84 | Jessore | 289 | Moulvibazar | 191 | Rangamati | 73 |
| 6 | 2223 | Cox-s Bazar | 47 | Jha | 211 | Munshiganj | 2 | Rangpur | 144 |
| 7 | 2431 | Cox-s-Bazar | 58 | Jhalokati | 96 | Mymensingh | 203 | Sa | 53 |
| 8 | 2411 | Cumilla | 81 | Jhenaidah | 162 | Na | 323 | Satkhira | 115 |
| 9 | 2071 | Da | 402 | Joypurhat | 45 | Naogaon | 123 | Sha | 131 |
| A | 184 | DA | 141 | Ka | 188 | Narail | 60 | Shariatpur | 86 |
| Ba | 409 | Dha | 136 | Kha | 264 | Narayanganj | 83 | Sherpur | 85 |
| Bagerhat | 37 | Dhaka | 4947 | Khagrachari | 151 | Narsingdi | 101 | Sirajganj | 139 |
| Bagura | 126 | Dinajpur | 82 | Khulna | 566 | Natore | 184 | Sunamganj | 120 |
| Bandarban | 62 | E | 133 | Kishoreganj | 117 | Netrokona | 65 | Sylhet | 250 |
| Barguna | 70 | Faridpur | 57 | Kurigram | 113 | Nilphamari | 84 | Ta | 713 |
| Barisal | 217 | Feni | 141 | Kustia | 45 | Noakhali | 140 | Tangail | 292 |
| Bha | 306 | Ga | 2853 | La | 2137 | Nowabganj | 11 | Tha | 405 |
| Bhola | 105 | Gaibandha | 54 | Lakshmipur | 128 | Pa | 76 | THA | 179 |
| Brahmanbaria | 68 | Gazipur | 106 | Lalmonirhat | 102 | Pabna | 144 | Thakurgaon | 123 |
| Cha | 430 | Gha | 652 | Ma | 201 | panchagarh | 73 | U | 188 |

**Comment 5:** *The recognition pipeline should be explained better; left-to-right sorting may not be enough for Bangladeshi plate structure, two-line plates and city/metro/type/number order.*

**Response:**

We cordially thank the reviewer for this insightful comment. We have already explained the pipeline in detail in our manuscript, in section 3.4, "Proposed VLPDR Model Architecture." In this section, we have explained that three YOLO-based models have been cascaded, where 1^st^ model detects the vehicle, 2^nd^ model localizes the license plate, and the 3^rd^ mode recognize the license plate number.

The second concern in this comment is the simple left-to-right sorting of Bangladeshi license plates. We completely agree that Bangladeshi vehicle license plates contain a two-line structure in which the first line has the city, metropolitan, and vehicle type information, and the second line has the vehicle class number (2 digits) and a unique number (4 digits). So, we have revised our code arrangement to match the two-line structure of the Bangladeshi license plate. We have also revised the associated figures (Figs. 29 and 30), which show the changes. For your concern we have attached an example figure here too.

Fig. 02: Proposed Model Output with Two-line plate structure.

**Comment 6:** *The real-time claim is weak, because some hardware results are not real-time; authors should report FPS and clearly state whether Jetson GPU/TensorRT or only CPU was used.*

**Response:**

We have completely agreed with this comment that more evidence is needed to claim real-time performance. So, we have removed the phrase “real-time” from our manuscript.

**Comment 7:** *Experimental comparison is limited; authors should compare with OCR-based and YOLO-based baseline methods on the same test set, not only literature table.*

**Response:**

We really appreciate the reviewer's valuable comment. As suggested, we tested an OCR-based approach that combines License Plate Detection (LPD) for detecting license plates and easy OCR for recognizing Bangla license numbers. Then we have compared the results with our proposed cascaded model’s results. For this evaluation, we have considered 150 images used in external validation to enable better comparison.

Among the 150 images, the OCR approach only succeeded in extracting 23 almost correct license plate numbers. Table 2 presents the correct extraction from the OCR-based approach model. So, the OCR-based approach has only achieved 15.33% accuracy. However, our proposed cascaded model has achieved 95.33% accuracy (Exact Match). So, we have revised our manuscript and enriched it with this comparison result. The full result on 150 External Validated Images of the proposed cascaded model and OCR-based approach can be found in this link:

<https://github.com/prosensarker4/BLPD/tree/main/External%20Validated%20Images>

Table 2: OCR-based Almost Correct Predictions.

| Image Name | Original License (English) | Original License (Bangla) | Predicted License (Bangla) |
| --- | --- | --- | --- |
| 2-RVRND-Test.jpg | Natore Ha 127394 | নাটোর হ ১২৭৩৯৪ | নাটোর হ ১২-৭৩৯৪ |
| 7-RVRND-Test.jpg | Jessore La 126898 | যশোর ল ১২৬৮৯৮ | যশোর: ল ১২-৬৮৯৮ |
| 10-RVRND-Test.jpg | Sirajganj Na 110028 | সিরাজগঞ্জ ন ১১০০২৮ | সিরাজগঞ্জ ন ১১-০০২৮ |
| 24-RVRND-Test.jpg | Dhaka Metro Bha 111891 | ঢাকা মেট্রো ভ ১১১৮৯১ | ঢাকা মেট্রো ভ ১১-১৮৯১ |
| 30-RVRND-Test.jpg | Dhaka Metro Ga 325029 | ঢাকা মেট্রো গ ৩২৫০২৯ | ঢাকা মেট্রো গ ৩২৪৫০২৯ |
| 35-RVRND-Test.jpg | Raj Metro Cha 510056 | রাজ মেট্রো চ ৫১০০৫৬ | রাজ মেট্রো-চ ৫১২০০৫৬ |
| 38-RVRND-Test.jpg | Noakhali La 110185 | নোয়াখালী ল ১১০১৮৫ | নোয়াখালী-ল ১১০১৮৫ |
| 39-RVRND-Test.jpg | Dhaka Metro Ga 211076 | ঢাকা মেট্রো গ ২১১০৭৬ | ঢাকা মেট্রো গ ২১-১০৭৬ |
| 45-RVRND-Test.jpg | Khulna La 112916 | খুলনা ল ১১২৯১৬ | খুলনা: ল ; ১১-২৯১৬ |
| 47-RVRND-Test.jpg | Pabna Ha 156366 | পাবনা হ ১৫৬৩৬৬ | পাবনা- হ ১৫-৬৩৬৬ |
| 61-RVRND-Test.jpg | Khagrachari Ha 113361 | খাগড়াছড়ি হ ১১৩৩৬১ | খাগড়াছড়ি: হ ১১-৩৩৬১ |
| 65-RVRND-Test.jpg | Khulna Metro Ka 110036 | খুলনা মেট্রো ক ১১০০৩৬ | খুলনা মে্ট্রো ক ১১২০০৩৬ |
| 71-RVRND-Test.jpg | Dhaka Metro Ga 318435 | ঢাকা মেট্রো গ ৩১৮৪৩৫ | ঢাকা খেট্রো গ ৩১-৮৪৩৫ |
| 93-RVRND-Test.jpg | Dhaka Metro Ga 324351 | ঢাকা মেট্রো গ ৩২৪৩৫১ | ঢাকা মেট্রো-গ ৩২-৪৩৫১ |
| 96-RVRND-Test.jpg | Dhaka Metro Ba 121158 | ঢাকা মেট্রো ব ১২১১৫৮ | ঢাকা মেট্রো-ব ১১ ১১৫৮ |
| 99-RVRND-Test.jpg | Dhaka Metro Gha 137061 | ঢাকা মেট্রো ঘ ১৩৭০৬১ | ঢাক] ফেট্রো ঘ [১৩৪৭0৬১\| |
| 100-RVRND-Test.jpg | Raj Metro Cha 510056 | রাজ মেট্রো চ ৫১০০৫৬ | রাজ মেট্রো চ ৫১-০০৫৬ |
| 107-RVRND-Test.jpg | Dhaka Metro Ba 114511 | ঢাকা মেট্রো ব ১১৪৫১১ | ঢাকা মেট্রো-ব ১১-৪৫১১ |
| 119-RVRND-Test.jpg | Thakurgaon Ha 129497 | ঠাকুরগাঁও হ ১২৯৪৯৭ | ঠাকরগাও: হ ১২ ৯৪৯৭ |
| 126-RVRND-Test.jpg | Dhaka Metro Bha 111981 | ঢাকা মেট্রো ভ ১১১৯৮১ | ঢাকা মেট্রো-ভ ১১-১৯৮১ |
| 128-RVRND-Test.jpg | Barguna La 110726 | বরগুনা ল ১১০৭২৬ | বরগুণা: ল ১১-০৭২৬ |
| 143-RVRND-Test.jpg | Lakshmipur Ha 120617 | লক্ষ্মীপুর হ ১২০৬১৭ | লক্ষ্মীপূর হ ১২-০৬১৭ |
| 147-RVRND-Test.jpg | Moulvibazar Ha 111067 | মৌলভীবাজার হ ১১১০৬৭ | মীলভীবজার- হ ১১-১০৬৭ |

So, Table 3 presents the comparison between the proposed cascaded model and the ORC-based approach results:

Table 3: Comparison between the Proposed Cascaded Model and ORC-based Approach Results

| Model | Images | Correct | Incorrect | Accuracy |
| --- | --- | --- | --- | --- |
| Proposed Cascaded Model | 150 | 143 | 7 | 95.33% |
| OCR-based Model | 150 | 23 | 127 | 15.33% |

**Comment 8:** *The authors should also read and discuss “Real-Time Theft Detection in Urban Surveillance: A Comparative Analysis of YOLO-Based Approach”, because it is related with YOLO-based urban surveillance, real-time deployment and comparative YOLO evaluation.*

**Response:**

Thank you for your suggestion. We have incorporated this article into our introduction section (line 66) to enrich the quality of our manuscript. Please find the reference 28.

**Comment 9:** *Generic YOLO theory part is too long, but implementation details are missing; authors should shorten basic YOLO explanation and add hyperparameters, augmentation, confidence threshold and NMS threshold.*

**Response:**

Thank you for your technical comment. We have revised our manuscript with a table (Table 5) that provides the experimental settings. For your convenience, we have shared the table below as a snip.

**Comment 10:** *English should be revised; phrases like “In this thesis” and repeated “has been” style should be corrected for journal paper format.*

**Response:**

Thank you for your comment. We have revised the phrase “*In this thesis*” and the use of excessive “*has been*” in your revised manuscript.
