# Supplementary material for: Cascaded YOLO-based approach for Bangladeshi license plate identification and number recognition
Source: PLoS One. 2026 Aug 10;21(8):e0355858. doi: 10.1371/journal.pone.0355858 (PMC13456284; doi:10.1371/journal.pone.0355858)
Supplement: Peer review history

# Peer review: Decision Letter 0

**DOI:** 10.1371/journal.pone.0355858.r001

**Authors:** Mustafa Hikmet Bilgehan Ucar

2 Jun 2026

PONE-D-26-12762

Cascaded YOLO-based approach for Bangladeshi license plate identification and number recognition

PLOS One

Dear Dr. Alam,

Thank you for submitting your manuscript to PLOS ONE. After careful consideration, we feel that it has merit but does not fully meet PLOS ONE’s publication criteria as it currently stands. Therefore, we invite you to submit a revised version of the manuscript that addresses the points raised during the review process.

Please submit your revised manuscript by Jul 17 2026 11:59PM. If you will need more time than this to complete your revisions, please reply to this message or contact the journal office at plosone@plos.org. When you're ready to submit your revision, log on to https://www.editorialmanager.com/pone/ and select the 'Submissions Needing Revision' folder to locate your manuscript file.

Please include the following items when submitting your revised manuscript:

- A letter that responds to each point raised by the academic editor and reviewer(s). You should upload this letter as a separate file labeled 'Response to Reviewers'.
- A marked-up copy of your manuscript that highlights changes made to the original version. You should upload this as a separate file labeled 'Revised Manuscript with Track Changes'.
- An unmarked version of your revised paper without tracked changes. You should upload this as a separate file labeled 'Manuscript'.

If you would like to make changes to your financial disclosure, please include your updated statement in your cover letter. Guidelines for resubmitting your figure files are available below the reviewer comments at the end of this letter.

If applicable, we recommend that you deposit your laboratory protocols in protocols.io to enhance the reproducibility of your results. Protocols.io assigns your protocol its own identifier (DOI) so that it can be cited independently in the future. For instructions see: https://journals.plos.org/plosone/s/submission-guidelines#loc-laboratory-protocols. Additionally, PLOS ONE offers an option for publishing peer-reviewed Lab Protocol articles, which describe protocols hosted on protocols.io. Read more information on sharing protocols at https://plos.org/protocols?utm_medium=editorial-email&utm_source=authorletters&utm_campaign=protocols.

As the corresponding author, your ORCID iD is verified in the submission system and will appear in the published article. PLOS supports the use of ORCID, and we encourage all coauthors to register for an ORCID iD and use it as well. Please encourage your coauthors to verify their ORCID iD within the submission system before final acceptance, as unverified ORCID iDs will not appear in the published article. *Only*  the individual author can complete the verification step; PLOS staff *cannot*  verify ORCID iDs on behalf of authors.

We look forward to receiving your revised manuscript.

Kind regards,

Prof. Mustafa Hikmet Bilgehan Ucar, Ph.D.

Academic Editor

PLOS One

Journal Requirements:

When submitting your revision, we need you to address these additional requirements.

1.Please ensure that your manuscript meets PLOS ONE's style requirements, including those for file naming. The PLOS ONE style templates can be found at

https://journals.plos.org/plosone/s/file?id=wjVg/PLOSOne_formatting_sample_main_body.pdf and

https://journals.plos.org/plosone/s/file?id=ba62/PLOSOne_formatting_sample_title_authors_affiliations.pdf

2. Please note that PLOS One has specific guidelines on code sharing for submissions in which author-generated code underpins the findings in the manuscript. In these cases, we expect all author-generated code to be made available without restrictions upon publication of the work. Please review our guidelines at https://journals.plos.org/plosone/s/materials-and-software-sharing#loc-sharing-code and ensure that your code is shared in a way that follows best practice and facilitates reproducibility and reuse.

3. We note that the grant information you provided in the ‘Funding Information’ and ‘Financial Disclosure’ sections do not match.

When you resubmit, please ensure that you provide the correct grant numbers for the awards you received for your study in the ‘Funding Information’ section.

4. We note that your Data Availability Statement is currently as follows: “All relevant data are within the manuscript and its Supporting Information files”

Please confirm at this time whether or not your submission contains all raw data required to replicate the results of your study. Authors must share the “minimal data set” for their submission. PLOS defines the minimal data set to consist of the data required to replicate all study findings reported in the article, as well as related metadata and methods (https://journals.plos.org/plosone/s/data-availability#loc-minimal-data-set-definition).

For example, authors should submit the following data:

- The values behind the means, standard deviations and other measures reported;

- The values used to build graphs;

- The points extracted from images for analysis.

Authors do not need to submit their entire data set if only a portion of the data was used in the reported study.

If your submission does not contain these data, please either upload them as Supporting Information files or deposit them to a stable, public repository and provide us with the relevant URLs, DOIs, or accession numbers. For a list of recommended repositories, please see https://journals.plos.org/plosone/s/recommended-repositories.

If there are ethical or legal restrictions on sharing a de-identified data set, please explain them in detail (e.g., data contain potentially sensitive information, data are owned by a third-party organization, etc.) and who has imposed them (e.g., an ethics committee). Please also provide contact information for a data access committee, ethics committee, or other institutional body to which data requests may be sent. If data are owned by a third party, please indicate how others may request data access.

5. If the reviewer comments include a recommendation to cite specific previously published works, please review and evaluate these publications to determine whether they are relevant and should be cited. There is no requirement to cite these works unless the editor has indicated otherwise.

Additional Editor Comments:

The manuscript presents a cascaded YOLO-based approach for Bangladeshi license plate detection and number recognition. The application area is relevant, and the attempt to recognize Bangladeshi license plate components through a 104-class framework without relying on a separate OCR stage is a potentially useful aspect of the study. However, several important issues need to be addressed before the manuscript can be considered further.

The following revisions are required:

1. The novelty and contribution of the study should be clarified more explicitly. A cascaded YOLO-based framework is not, by itself, sufficiently novel. The manuscript should clearly explain what is technically new in the proposed 104-class Bangladeshi license plate recognition approach, especially in comparison with existing YOLO-based and OCR-based license plate recognition systems.

2. The choice of YOLOv12 should be technically justified. The manuscript should include comparative experiments with relevant YOLO versions, such as YOLOv8, YOLOv9, YOLOv10, or YOLOv11, using the same dataset split, training protocol, evaluation metrics, and hardware conditions.

3. The experimental comparison should be strengthened. Comparisons should not rely only on literature-level summaries. The authors should compare the proposed model with OCR-based and YOLO-based baseline methods on the same test set wherever possible.

4. The dataset description requires substantial improvement. The manuscript should provide clearer information on image sources, camera conditions, lighting conditions, day/night images, weather variation, vehicle types, geographical diversity, city coverage, dataset splits, class distribution, and external validation images.

5. The class imbalance in Dataset 02 should be discussed in detail. Per-class precision, recall, F1-score, and confusion matrices should be provided, particularly for visually similar Bangla characters and classes that may be difficult to distinguish.

6. The recognition pipeline should be explained more rigorously. The manuscript should clarify whether simple left-to-right sorting is sufficient for Bangladeshi plate structures, including two-line plates and the ordering of city, metro/type, and number components.

7. The real-time performance claim should be revised or supported with stronger evidence. The authors should report FPS, latency, model size, computational cost, and hardware details. It should be clearly stated whether inference was performed using CPU, GPU, Jetson GPU, TensorRT, or other acceleration settings. If some reported results are not real-time, the claim should be moderated accordingly.

8. Table 1 should be revised. It should include, for each reviewed study, the method used, dataset name, dataset size, language or plate type, main advantages, main disadvantages, and key performance metrics. The comparison should also discuss relevant works in other languages or plate systems to better contextualize the efficiency of the proposed model.

9. The manuscript should include more recent references where relevant, including studies published in 2026 if directly related to license plate recognition, YOLO-based recognition, OCR-free recognition, or real-time intelligent transportation/urban surveillance applications.

10. The general YOLO background section should be shortened. Instead of providing an extended generic explanation of YOLO, the manuscript should give more implementation-specific details, including hyperparameters, augmentation strategy, image size, batch size, number of epochs, optimizer, learning rate, confidence threshold, NMS threshold, early stopping criteria, and training/validation settings.

11. The conclusion should be revised to include a concise paragraph summarizing future work. This may include broader external validation, deployment under challenging environmental conditions, improved treatment of class imbalance, and comparison with lightweight real-time models.

12. The English language and journal style should be revised carefully. Phrases such as “In this thesis” and repeated or awkward constructions such as excessive “has been” usage should be corrected. The manuscript should be presented consistently as a journal article.

The revised manuscript should include a detailed response letter explaining how each reviewer comment has been addressed. Where a requested experiment or comparison cannot be performed, a clear technical justification should be provided.

[Note: HTML markup is below. Please do not edit.]

Reviewer's Responses to Questions

**Comments to the Author**

1. Is the manuscript technically sound, and do the data support the conclusions?

The manuscript must describe a technically sound piece of scientific research with data that supports the conclusions. Experiments must have been conducted rigorously, with appropriate controls, replication, and sample sizes. The conclusions must be drawn appropriately based on the data presented.

Reviewer #1: Yes

Reviewer #2: Partly

**********

2. Has the statistical analysis been performed appropriately and rigorously?

Reviewer #1: Yes

Reviewer #2: Yes

**********

3. Have the authors made all data underlying the findings in their manuscript fully available?

The PLOS Data policy requires authors to make all data underlying the findings described in their manuscript fully available without restriction, with rare exception (please refer to the Data Availability Statement in the manuscript PDF file). The data should be provided as part of the manuscript or its supporting information, or deposited to a public repository. For example, in addition to summary statistics, the data points behind means, medians and variance measures should be available. If there are restrictions on publicly sharing data—e.g. participant privacy or use of data from a third party—those must be specified.

Reviewer #1: Yes

Reviewer #2: Yes

**********

4. Is the manuscript presented in an intelligible fashion and written in standard English?

PLOS ONE does not copyedit accepted manuscripts, so the language in submitted articles must be clear, correct, and unambiguous. Any typographical or grammatical errors should be corrected at revision, so please note any specific errors here.

Reviewer #1: Yes

Reviewer #2: Yes

**********

5. Review Comments to the Author

Please use the space provided to explain your answers to the questions above. You may also include additional comments for the author, including concerns about dual publication, research ethics, or publication ethics. (Please upload your review as an attachment if it exceeds 20,000 characters)

Reviewer #1: 1- Table 1 did not mention the advantages and disadvantages of each method used in each study, in addition to the name and size of the dataset used.

2- Why wasn't the submitted work compared with similar works in other languages to ensure the efficiency of the proposed model?

3-Future actions can be summarized in a paragraph and included in the conclusions.

4- Adding other references published in 2026

Reviewer #2: The manuscript proposes a cascaded YOLO-based framework for Bangladeshi license plate detection and recognition. The topic is useful and the 104-class Bangla plate recognition without OCR is interesting, but some points should be revised before publication.

1. The novelty should be explained more clear; cascaded YOLO structure is not very new, so authors should emphasize the real contribution of 104-class Bangladeshi plate recognition without OCR.

2. The reason of selecting YOLOv12 should be justified, and comparison with YOLOv8, YOLOv9, YOLOv10 or YOLOv11 should be added under same dataset and training settings.

3. Dataset description and validation should be improved; image sources, camera conditions, day/night, weather, vehicle types, split details and more external images from different cities should be given.

4. Dataset 02 has visible class imbalance, but this is not discussed enough; per-class precision, recall and confusion matrix should be given especially for similar Bangla characters.

5. The recognition pipeline should be explained better; left-to-right sorting may not be enough for Bangladeshi plate structure, two-line plates and city/metro/type/number order.

6. The real-time claim is weak, because some hardware results are not real-time; authors should report FPS and clearly state whether Jetson GPU/TensorRT or only CPU was used.

7. Experimental comparison is limited; authors should compare with OCR-based and YOLO-based baseline methods on the same test set, not only literature table.

8. The authors should also read and discuss “Real-Time Theft Detection in Urban Surveillance: A Comparative Analysis of YOLO-Based Approach”, because it is related with YOLO-based urban surveillance, real-time deployment and comparative YOLO evaluation.

9. Generic YOLO theory part is too long, but implementation details are missing; authors should shorten basic YOLO explanation and add hyperparameters, augmentation, confidence threshold and NMS threshold.

10. English should be revised; phrases like “In this thesis” and repeated “has been” style should be corrected for journal paper format.

**********

6. PLOS authors have the option to publish the peer review history of their article (what does this mean?). If published, this will include your full peer review and any attached files.

If you choose “no”, your identity will remain anonymous but your review may still be made public.

**Do you want your identity to be public for this peer review?**  For information about this choice, including consent withdrawal, please see our Privacy Policy.

Reviewer #1: No

Reviewer #2: No

**********

[NOTE: If reviewer comments were submitted as an attachment file, they will be attached to this email and accessible via the submission site. Please log into your account, locate the manuscript record, and check for the action link "View Attachments". If this link does not appear, there are no attachment files.]

To ensure your figures meet our technical requirements, please review our figure guidelines: https://journals.plos.org/plosone/s/figures

You may also use PLOS’s free figure tool, NAAS, to help you prepare publication quality figures: https://journals.plos.org/plosone/s/figures#loc-tools-for-figure-preparation.

NAAS will assess whether your figures meet our technical requirements by comparing each figure against our figure specifications.

# Author comment: Author response to Decision Letter 1

**DOI:** 10.1371/journal.pone.0355858.r002

8 Jul 2026

As per the reviewers' and editor's comments, we have attached a Response to Reviewers.docx file as an attachment.

Attachment Submitted filename: Response to Reviewers.docx

# Peer review: Decision Letter 1

**DOI:** 10.1371/journal.pone.0355858.r003

**Authors:** Mustafa Hikmet Bilgehan Ucar

27 Jul 2026

Cascaded YOLO-based approach for Bangladeshi license plate identification and number recognition

PONE-D-26-12762R1

Dear Dr. Alam,

We’re pleased to inform you that your manuscript has been judged scientifically suitable for publication and will be formally accepted for publication once it meets all outstanding technical requirements.

Within one week, you’ll receive an e-mail detailing the required amendments. When these have been addressed, you’ll receive a formal acceptance letter and your manuscript will be scheduled for publication.

An invoice will be generated when your article is formally accepted. Please note, if your institution has a publishing partnership with PLOS and your article meets the relevant criteria, all or part of your publication costs will be covered. Please make sure your user information is up-to-date by logging into Editorial Manager at Editorial Manager® and clicking the ‘Update My Information' link at the top of the page. For questions related to billing, please contact billing support.

If your institution or institutions have a press office, please notify them about your upcoming paper to help maximize its impact. If they’ll be preparing press materials, please inform our press team as soon as possible -- no later than 48 hours after receiving the formal acceptance. Your manuscript will remain under strict press embargo until 2 pm Eastern Time on the date of publication. For more information, please contact onepress@plos.org.

Kind regards,

Mustafa Hikmet Bilgehan Ucar, Ph.D.

Academic Editor

PLOS One

Additional Editor Comments (optional):

After considering the reviewers’ evaluations and my own assessment, I am pleased to inform you that your manuscript is accepted for publication.

The reviewers found the study to address a relevant application area and recognized the value of the proposed cascaded YOLO-based approach for Bangladeshi license plate identification and number recognition. The OCR-free 104-class recognition framework is a useful aspect of the work, and the manuscript was considered technically sound overall. Congratulations on the acceptance of your manuscript.

Reviewers' comments:

Reviewer's Responses to Questions

**Comments to the Author**

1. If the authors have adequately addressed your comments raised in a previous round of review and you feel that this manuscript is now acceptable for publication, you may indicate that here to bypass the “Comments to the Author” section, enter your conflict of interest statement in the “Confidential to Editor” section, and submit your "Accept" recommendation.

Reviewer #1: (No Response)

Reviewer #2: All comments have been addressed

**********

2. Is the manuscript technically sound, and do the data support the conclusions?

The manuscript must describe a technically sound piece of scientific research with data that supports the conclusions. Experiments must have been conducted rigorously, with appropriate controls, replication, and sample sizes. The conclusions must be drawn appropriately based on the data presented.

Reviewer #1: (No Response)

Reviewer #2: Yes

**********

3. Has the statistical analysis been performed appropriately and rigorously?

Reviewer #1: (No Response)

Reviewer #2: N/A

**********

4. Have the authors made all data underlying the findings in their manuscript fully available?

The PLOS Data policy requires authors to make all data underlying the findings described in their manuscript fully available without restriction, with rare exception (please refer to the Data Availability Statement in the manuscript PDF file). The data should be provided as part of the manuscript or its supporting information, or deposited to a public repository. For example, in addition to summary statistics, the data points behind means, medians and variance measures should be available. If there are restrictions on publicly sharing data—e.g. participant privacy or use of data from a third party—those must be specified.

Reviewer #1: (No Response)

Reviewer #2: Yes

**********

5. Is the manuscript presented in an intelligible fashion and written in standard English?

PLOS ONE does not copyedit accepted manuscripts, so the language in submitted articles must be clear, correct, and unambiguous. Any typographical or grammatical errors should be corrected at revision, so please note any specific errors here.

Reviewer #1: (No Response)

Reviewer #2: Yes

**********

6. Review Comments to the Author

Please use the space provided to explain your answers to the questions above. You may also include additional comments for the author, including concerns about dual publication, research ethics, or publication ethics. (Please upload your review as an attachment if it exceeds 20,000 characters)

Reviewer #1: (No Response)

Reviewer #2: The revised manuscript has been substantially improved compared with the previous version. The authors have carefully addressed the reviewers’ comments and provided clear point-by-point responses in the rebuttal document. The methodology, experimental evaluation, presentation of the results, and overall clarity of the manuscript have been significantly strengthened. In my opinion, the revised version is now suitable for publication. I therefore recommend acceptance.

**********

7. PLOS authors have the option to publish the peer review history of their article (what does this mean?). If published, this will include your full peer review and any attached files.

If you choose “no”, your identity will remain anonymous but your review may still be made public.

**Do you want your identity to be public for this peer review?**  For information about this choice, including consent withdrawal, please see our Privacy Policy.

Reviewer #1: No

Reviewer #2: No

**********

# Editor report: Acceptance letter

**DOI:** 10.1371/journal.pone.0355858.r004

**Authors:** Mustafa Hikmet Bilgehan Ucar

PONE-D-26-12762R1

PLOS One

Dear Dr. Alam,

I'm pleased to inform you that your manuscript has been deemed suitable for publication in PLOS One. Congratulations! Your manuscript is now being handed over to our production team.

At this stage, our production department will prepare your paper for publication. This includes ensuring the following:

* All references, tables, and figures are properly cited

* All relevant supporting information is included in the manuscript submission,

* There are no issues that prevent the paper from being properly typeset

You will receive further instructions from the production team, including instructions on how to review your proof when it is ready. Please keep in mind that we are working through a large volume of accepted articles, so please give us a few days to review your paper and let you know the next and final steps.

Lastly, if your institution or institutions have a press office, please let them know about your upcoming paper now to help maximize its impact. If they'll be preparing press materials, please inform our press team within the next 48 hours. Your manuscript will remain under strict press embargo until 2 pm Eastern Time on the date of publication. For more information, please contact onepress@plos.org.

You will receive an invoice from PLOS for your publication fee after your manuscript has reached the completed accept phase. If you receive an email requesting payment before acceptance or for any other service, this may be a phishing scheme. Learn how to identify phishing emails and protect your accounts at https://explore.plos.org/phishing.

If we can help with anything else, please email us at customercare@plos.org.

Thank you for submitting your work to PLOS ONE and supporting open access.

Kind regards,

PLOS ONE Editorial Office Staff

on behalf of

Dr. Mustafa Hikmet Bilgehan Ucar

Academic Editor

PLOS One
